# Applying Ultrasound to Mechanically and Noninvasively Sensitize Prostate Tumors to TRAIL‐Mediated Apoptosis

**DOI:** 10.1002/advs.202412995

**Published:** 2025-02-20

**Authors:** Abigail R. Fabiano, Malachy W. Newman, Jenna A. Dombroski, Schyler J. Rowland, Samantha V. Knoblauch, Jiro Kusunose, Katherine N. Gibson‐Corley, Benjamin G. Kaufman, Liqin Ren, Charles F. Caskey, Michael R. King

**Affiliations:** ^1^ Department of Biomedical Engineering Vanderbilt University Nashville TN 37235 USA; ^2^ Department of Bioengineering Rice University Houston TX 77005 USA; ^3^ Vanderbilt University Institute of Imaging Science Vanderbilt University Medical Center Nashville TN 37235 USA; ^4^ Department of Pathology Microbiology and Immunology Division of Comparative Medicine Vanderbilt University Medical Center Nashville TN 37235 USA; ^5^ Department of Radiology and Radiological Sciences Vanderbilt University Nashville TN 37235 USA

**Keywords:** mechanotransduction, therapeutic ultrasound, noninvasive, mechanotherapy, synergistic

## Abstract

Non‐surgical and safe prostate cancer (PCa) therapies are in demand. Soluble tumor necrosis factor (TNF‐α) related apoptosis inducing ligand (TRAIL), a cancer‐specific drug, shows preclinical efficacy but has a short circulation half‐life. This research has shown that physiological fluid shear stress activates mechanosensitive ion channels (MSCs), such as Piezo1, enhancing TRAIL‐mediated apoptosis in cancer cells. Herein, noninvasive, focal ultrasound (FUS) is implemented to augment the pro‐apoptotic effects of TRAIL. Using thermally safe FUS parameters, it is observed that TRAIL sensitivity increases with higher FUS pressure in PCa cells, mediated by Piezo1. This is confirmed by examining the effects of calcium chelation, MSC inhibitors, and *PIEZO* knockdown. In vivo, a multi‐dose study with 10 min FUS exposure shows that 0 and 4‐h intervals between TRAIL and FUS significantly reduce tumor burden, with an increase in apoptosis evident by enhanced cleaved‐caspase 3 expression. This mechanotherapy offers a clinically translatable approach by utilizing widely available FUS technology, applicable to treat additional cancer types.

## Graphical abstract

This study explores the application of therapeutic, focal ultrasound to noninvasively enhance the anti‐cancer effects of Tumor necrosis factor‐related apoptosis‐inducing ligand (TRAIL) as a targeted treatment for prostate cancer. This is achieved by mechanically activating mechanosensitive ion channels within cancer cells. The findings emphasize promising, clinically translatable approaches to locally treat cancer and improve patient outcomes, while leveraging widely available technologies to maximize therapeutic impact.

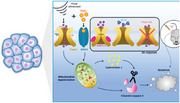

## 1 Introduction

Each year, it is estimated that there are ten million cancer‐related deaths worldwide.^[^ ^1^ ^]^ Targeting cancer cells at the primary tumor is imperative to prevent the formation of distant metastatic lesions, which can form when tumor cells escape the primary tumor microenvironment (TME) and survive transit through the circulatory system. Once metastasis occurs, the survival rate is significantly reduced. In men, prostate cancer (PCa) is the second leading cause of death in the United States and is the most frequently diagnosed cancer in 112 different countries.^[^ ^1^, ^2^ ^]^ Androgen deprivation therapy is commonly used as a standard‐of‐care treatment for advanced or recurring PCa; but this may induce severe side‐effects, including fatigue and bone fracture.^[^ ^2^, ^3^ ^]^ Radiation therapy provides another non‐surgical means to treat PCa; however, radiation can damage the tissue and neurovascular bundles surrounding the prostate.^[^ ^4^ ^]^ Focused ultrasound (FUS)‐based therapies are gaining more attention due to their ability to specifically target tumor regions, leaving surrounding tissue undamaged. For instance, high‐intensity focused ultrasound (HIFU) offers a relatively quick, minimally invasive treatment, that is becoming an integral part of many combination therapies to induce cancer cell apoptosis via hyperthermia while reducing relapse, in contrast to low‐intensity focused ultrasound (LIFU) which is more commonly used to enhance drug delivery in disease treatment.^[^ ^5^, ^6^, ^7^, ^8^, ^9^ ^]^ HIFU is now FDA‐approved to treat PCa, but has potential to cause adverse effects such as skin burns and urinary complications. Thus, development of a less destructive FUS therapy to treat PCa is highly desirable.^[^ ^10^ ^]^

Tumor necrosis factor (TNF‐α) related apoptosis inducing ligand (TRAIL) is an anti‐cancer drug that selectively causes apoptosis in tumor cells through binding to death receptors 4 and 5 (DR4/5) to induce extrinsic apoptosis, while sparing healthy cells.^[^ ^11^, ^12^, ^13^ ^]^ The first soluble recombinant version of naturally occurring TRAIL was dulanermin, which has reached Phase II and III clinical trials and resulted in only a small subset of cancer patients who benefited from the therapy.^[^ ^14^, ^15^, ^16^ ^]^ Dulanermin has been tested alone intravenously or combined with additional chemotherapies such as cisplatin.^[^ ^16^, ^17^ ^]^ However, dulanermin exhibited reduced efficacy due to a brief half‐life in circulation (≈30 min) upon intravenous injection, and adverse effects such as fatigue, nausea and fever in some patients.^[^ ^18^, ^19^, ^20^ ^]^ Most agonistic antibodies for TRAIL receptors have only entered Phase II clinical trials due to their relatively weak ability to induce receptor aggregation for downstream apoptosis, and potential adverse events.^[^ ^16^, ^21^ ^]^ Formulating anti‐cancer therapies that can be intratumorally inoculated and remain effective, would offer advantages to reduce off‐target toxicity and lower the dose of therapeutic regimen required to induce an anti‐tumor response.^[^ ^22^ ^]^

Our previous research showed that combination treatment of cancer cells with TRAIL and physiologically relevant fluid shear stress (FSS) enhanced TRAIL‐mediated cell death and TRAIL sensitization.^[^ ^12^, ^23^, ^24^, ^25^, ^26^ ^]^ Further examination of this mechanism determined that the mechanosensitive ion channel (MSC), Piezo1 (Piezo Type Mechanosensitive Ion Channel Component 1), was mechanically opened and activated in response to the membrane tension induced by the shear forces. This leads to calcium (Ca^2+^) influx into the cells to promote the intrinsic apoptosis pathway synergistically with the downstream effects of TRAIL binding to DR4/5.^[^ ^12^ ^]^ We also previously showed that docetaxel and cabazitaxel sensitize PC3 and TRAIL‐resistant DU145 cells to TRAIL‐mediated apoptosis through the induction of endoplasmic reticulum stress.^[^ ^27^ ^]^ We created TRAIL‐coated leukocytes, TRAIL‐conjugated liposomes that can bind NK cells, and dual affinity nanoparticles functionalized with TRAIL, E‐selectin, and anti‐cell surface vimentin half‐antibody; which have all been shown to enhance circulating tumor cell (CTC) apoptosis under shear flow conditions.^[^ ^24^, ^25^, ^26^, ^28^ ^]^ Research has also shown how highly tunable, photomagenetically powered nanomachines are able to enhance the mechanical destruction of cancer cells.^[^ ^29^ ^]^ Despite the notable success with these technologies, one limitation is that they require the production and use of more complex formulations and vehicles to achieve targeted TRAIL delivery, and are leveraged toward targeting tumor cells in the circulatory and lymphatic systems. Additionally, more than 95% of encapsulated anti‐cancer drugs that are administered intravenously have been found to accumulate in other organs, introducing unwanted toxicity.^[^ ^30^, ^31^ ^]^ Taken together, the field is still lacking a straightforward and effective approach to treat the primary tumor to help prevent metastases by employing soluble TRAIL as a localized therapeutic.

Here we present a mechanotherapy that uses therapeutic, FUS as a mechanical stimulus to influence Piezo1 and enhance TRAIL‐mediated apoptosis in PCa cells. This provides a noninvasive anti‐cancer therapy that could be adapted to treat other cancers as well. One advantage to this treatment is that FUS is a widely used and readily available technology. With the enhanced therapeutic effects from the TRAIL treatment, the goal is to also reduce and refine the intensity of the ultrasonic parameters without causing thermal ablation to offer a harmless and more promising alternative. Here, we implement focal FUS technology with local delivery of soluble, recombinant TRAIL to target tumor cells. Previous research testing the effects of US with TRAIL has developed co‐encapsulation of doxorubicin and TRAIL in US‐responsive microbubbles to enhance apoptosis, which would still require the need for complex vehicle construction.^[^ ^32^ ^]^ US‐targeted microbubble destruction has also been shown to reverse the epithelial to mesenchymal transition (EMT) in breast cancer cells, thereby reducing metastatic ability.^[^ ^33^ ^]^ The combined use of US contrast agents with taurolidine, TRAIL and US exposure has also been explored; however, this therapy has not yet been tested in vivo.^[^ ^34^ ^]^

Our results demonstrate that FUS pressure positively correlates with the observed TRAIL sensitization in PCa cells, and that mechanoactivation of Piezo1 contributes significantly to the FUS+TRAIL‐induced apoptosis. In vivo models demonstrate the success of treating localized metastatic lesions through minimal FUS+TRAIL treatments via enhanced cell death through the synergistic intrinsic apoptosis pathway mediated by Piezo1 activation, while maintaining an unaltered EMT state. In this study, our goal is to develop a therapeutically relevant and safe method to induce cancer cell apoptosis in situ by taking advantage of cancer cell mechanotransduction. The pharmacokinetics of intratumorally administered therapeutics is not yet well understood and characterized, and these studies shed new light on how soluble TRAIL responds within the compact tumor environment.^[^ ^35^ ^]^ We further explore the mechanoresponse of PCa cells to FUS‐mediated therapy to provide methods that can overcome drug resistance in cancer and enhance therapeutic efficacy.

## 2 Results

### 2.1 Elevated Ultrasound Pressure Enhances TRAIL‐Mediated Apoptosis in Cancer Cells with Differential Metastatic Potential

**Figure 1:**
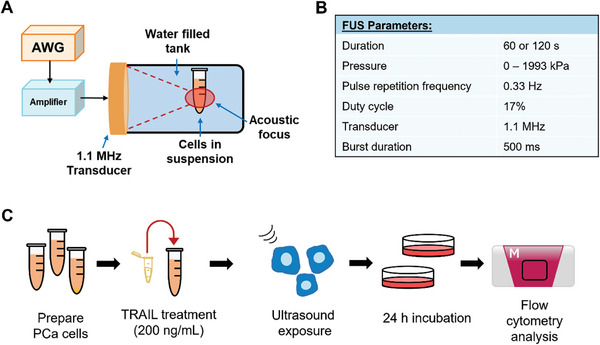
In vitro experimental overview. A) Ultrasound apparatus, B) parameter overview, and C) general process overview for in vitro experiments.

The in vitro FUS apparatus is shown in **Figure** **1**A. For the results discussed, all FUS parameters are consistent with those in Figure 1B unless otherwise noted and the general process overview for the in vitro experiments is shown in Figure 1C. LNCaP cells were derived from lymph node metastases and PC3 cells were derived from bone metastases, thus we first examined and compared the response of these cell lines to understand how exposure to FUS affects apoptosis in cells of varied metastatic potential.^[^ ^36^, ^37^ ^]^

**Figure 2:**
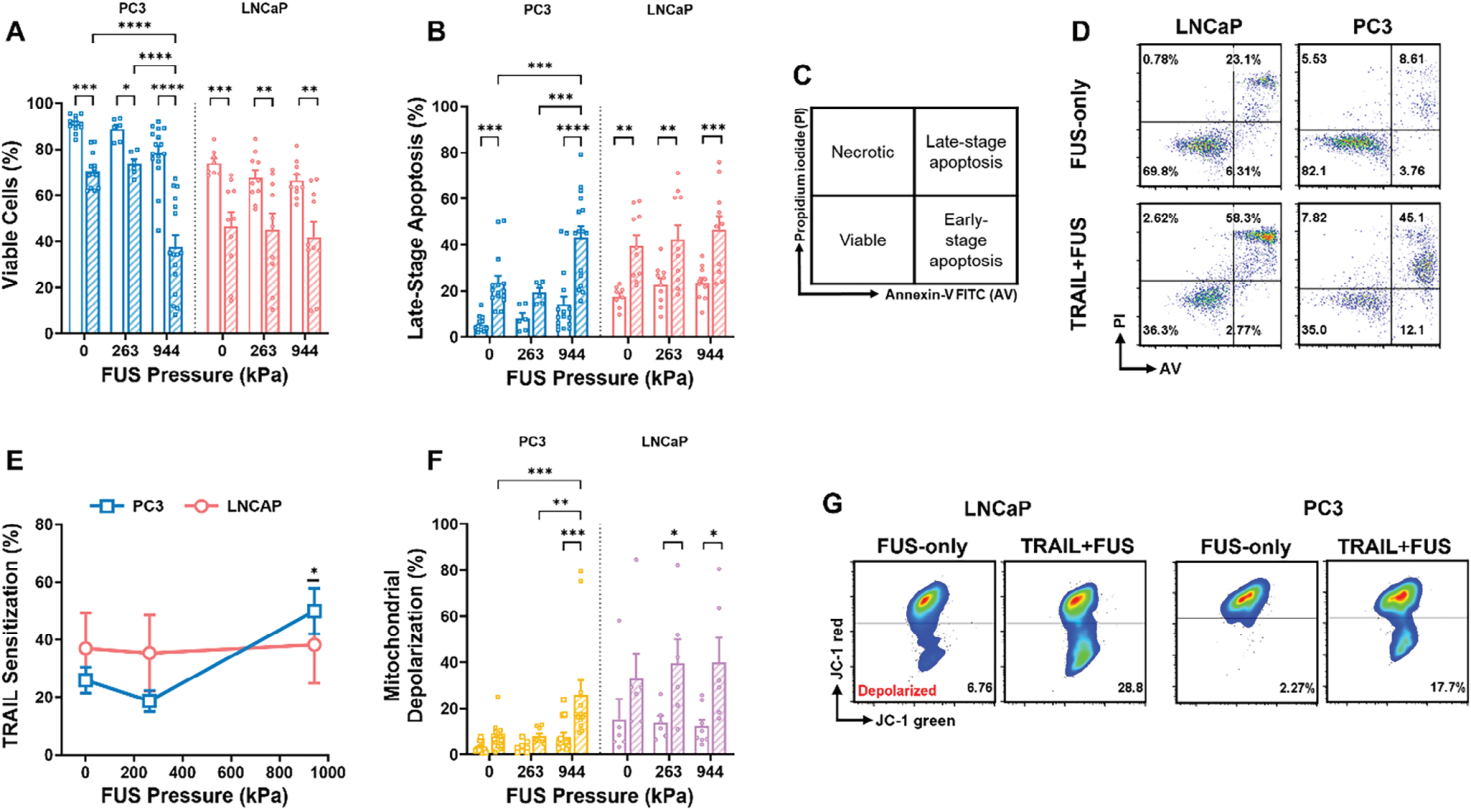
Effect of increased ultrasound pressure on LNCaP and PC3 cells. Summary data for the mean percentage of cells that are A) viable and B) in late‐stage apoptosis. C) Gating for AV/PI flow cytometry assay and D) representative AV/PI flow cytometry plots at 944 kPa. E) Summary data for the mean TRAIL sensitization (Equations (2) and (3)). F) Summary data for the mean percentage of depolarized mitochondria and G) representative JC‐1 flow cytometry plots at 944 kPa. *n* = 3–5 independent experiments; two‐way ANOVA for each cell line (A, B, F) and unpaired *t*‐test comparing the TRAIL sensitization at each pressure to samples with no ultrasound exposure (E). *p < 0.05, **p < 0.01, ***p < 0.005, ****p < 0.0001. Error bars represent mean ± SEM.

LNCaP and PC3 PCa cells were treated with 200 ng mL^−1^ of soluble, human TRAIL and then immediately exposed to low (263 kPa) or high (944 kPa) pressure FUS. 24 h after treatment, the degree of apoptosis of the PCa cells was examined using the Annexin‐V/propidium iodide (AV/PI) viability assay in flow cytometry. Cells positive for both AV and PI are in late‐stage apoptosis, and cells negative for both are considered viable. **Figure** **2** shows that as the FUS pressure increased, the TRAIL‐mediated apoptosis increased more notably in the PC3 cells (Figure 2A–D) in comparison to the LNCaP cells. Apoptosis in the TRAIL‐treated PC3 cells showed a 2.23‐fold increase when the pressure increased from 263 to 944 kPa. However, the LNCaP cells with or without FUS showed a plateau in sensitivity to TRAIL, still exhibiting significant increases in apoptosis compared to the 0, 263, and 944 kPa FUS‐only conditions (Figure 2B). The AV/PI flow cytometry plots were gated as shown in Figure 2C.

FUS‐induced TRAIL sensitization was calculated by subtracting the cell viability of the TRAIL‐treated group from its respective non‐TRAIL‐treated group, and then dividing by the viability of the non‐TRAIL‐treated condition (Equations (2) and (3)). TRAIL sensitization is a measure of the extent to which the FUS mechanical stimulus enhances the pro‐apoptotic effects of TRAIL.^[^ ^9^ ^]^ The TRAIL sensitization was highest for both cell lines at 944 kPa, reaching 38.3 ± 13.4% (SEM) for the LNCaPs and 49.9 ± 7.96% for the PC3s at 944 kPa, respectively (Figure 2E). The sensitivity of LNCaP cells to TRAIL remained constant and increased significantly for the PC3 cells at 944 kPa, likely due to the decreased vulnerability of LNCaP cells to TRAIL.^[^ ^38^ ^]^ There was a 1.03‐fold increase for the LNCaPs and 1.92‐fold increase for the PC3s compared to samples with no FUS exposure.

To examine the extent of apoptosis associated with the intrinsic pathway, the percentage of mitochondrial depolarization was quantified using the JC‐1 flow cytometry assay. A decrease in JC‐1 red fluorescence is indicative of monomeric, depolarized mitochondria.^[^ ^12^ ^]^ The mitochondrial depolarization for the LNCaP and PC3 cells is shown in Figure 2F,G. The highest depolarization for both cell lines was at 944 kPa for the TRAIL‐treated samples, reaching 40.0 ± 10.7% for the LNCaPs and 25.8 ± 6.48% for the PC3s. Elevated and nearly unchanged mitochondrial depolarization for the LNCaP cells at 0, 263, and 944 kPa FUS corroborates how the FUS fails to further enhance the pro‐apoptotic effects of TRAIL in these cells.^[^ ^38^ ^]^ These results encouraged us to further characterize the response of the PC3 cells to FUS+TRAIL treatment to investigate if we can enhance the therapeutic efficacy in these highly metastatic cells to enhance clinical relevance. Thus, we next employed 60 and 120 s of sonication at constant pressure (380 kPa), followed by exposure to pressures ranging from 263–1993 kPa at constant duration (60 s).

### 2.2 Synergistic Apoptosis in PC3 Cells Shows a Monotonic Dependence on Ultrasound Pressure

Our previous results demonstrate that LNCaP cells are more sensitive to FUS+TRAIL therapy at 263 and 944 kPa (Figure 2). We also confirmed that doubling the FUS duration at low pressure (380 kPa) had no effect on the TRAIL‐mediated apoptosis (Figure S1, Supporting Information) or mitochondrial depolarization (Figure S2, Supporting Information) in PC3 cells. These findings indicate that to achieve an effective dose of therapeutic FUS to treat highly metastatic cancer using this combination treatment, the mechanical index (MI) must be raised, which is a pressure‐dependent parameter used to assess the potential for cavitation during FUS procedures.^[^ ^39^, ^40^ ^]^ We also verified that there was no significant thermal effect from the FUS that is contributing to cell death. For 60 s of sonication the rise in temperature was 1.10 °C, and for a 90 s sonication the temperature rise was limited to 1.25 °C at 944 kPa (Figure S3, Supporting Information). Sonic treatments at lower pressure are expected to generate less heat. For diagnostic medical devices, temperature variations below 2 °C are generally considered safe.^[^ ^39^, ^40^, ^41^, ^42^ ^]^

**Figure 3:**
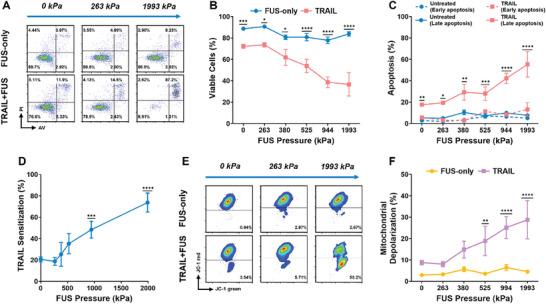
Intrinsic apoptosis is enhanced with increasing ultrasound pressure. A) Representative AV/PI flow cytometry plots for the summary data showing the mean percentage of B) viable cells, C) apoptotic cells, and D) TRAIL sensitization (Equations (2) and (3)). E) Representative JC‐1 flow cytometry plots and F) summary data for the mean percentage of depolarized mitochondria. PC3 cells were treated with FUS, TRAIL, or FUS+TRAIL for 60 s of sonication. Some of the experimental values at 263 and 944 kPa are the same used in Figure 2 and some of the same replicates at 380 kPa (60 s sonication) are those used in Figures S1 and S2 (Supporting Information). *n* = 3–5 independent experiments; two‐way ANOVA (B, C, F) and unpaired *t*‐test comparing the TRAIL sensitization at each pressure to the no‐FUS control (D). *p < 0.05, **p < 0.01, ***p < 0.005, ****p < 0.0001. Error bars represent mean ± SEM.

To further characterize the potential to induce apoptosis in a highly metastatic PCa cell line, a pressure‐dose curve was generated to determine how varying the peak pressure of the FUS pulse alters the therapeutic efficacy of TRAIL in vitro in response to the mechanical stimulus. PC3 cells were exposed to increasing FUS pressures ranging from 0–1993 kPa, with 60 s duration and a constant TRAIL concentration. As the FUS pressure increased, cellular viability 24 h after treatment significantly decreased and remained lower for the TRAIL‐treated conditions that were exposed to FUS (**Figure** **3**A,B). This is consistent with a significant increase in late‐stage apoptosis for the FUS+TRAIL‐treated cells (Figure 3A,C). Early‐stage apoptosis remained low for all conditions, likely because we examined the effects of the mechanotherapy 24 h after treatment. Cancer cells treated with TRAIL concentrations higher than 50 ng mL^−1^ have been shown to undergo maximal early apoptosis and depolarization before 24 h.^[^ ^12^, ^43^, ^44^, ^45^ ^]^ This is likely associated with the rapid execution of the caspase cascade once the executioner caspases are activated, leading to notable increases in late‐stage cell death.^[^ ^13^, ^45^, ^46^ ^]^ TRAIL sensitization for PC3 cells treated with FUS+TRAIL increased significantly from 20.5 ± 2.31% at 0 kPa to 73.7 ± 8.93% at 1993 kPa (Figure 3D), suggesting that the intensity of the FUS rather than the duration has a more pronounced effect on cancer cell apoptosis.

Mitochondrial depolarization was measured in PC3 cells 24 h after FUS+TRAIL treatment to measure the extent to which the intrinsic apoptosis pathway is active. As the FUS pressure increased, the mitochondrial depolarization increased significantly for the TRAIL‐treated conditions (Figure 3E,F). The percentage of depolarized mitochondria reached 28.8 ± 8.95% at 1993 kPa, increasing from just 8.76 ± 1.01% at 0 kPa. The similar trends seen in the late‐stage apoptosis and mitochondrial depolarization suggest intrinsic apoptosis due to mechanoactivation of Piezo1 induced by mechanical FUS forces acting on the cell membrane.

### 2.3 Inhibition of Piezo1 Diminishes Ultrasound‐Induced TRAIL‐Mediated Apoptosis

We used GsMTx‐4 to inhibit Piezo1, since this molecule is the only known pharmacological inhibitor of cationic MSCs (Figure S4, Supporting Information).^[^ ^47^, ^48^, ^49^, ^50^ ^]^ Previously in our lab we have shown that pre‐treatment of PC3 cells with GsMTx‐4 followed by exposure to low‐intensity FSS+TRAIL allowed for higher cell viability compared to the FSS+TRAIL condition.^[^ ^12^ ^]^ We have also seen that treatment of LN18 glioblastoma cells with GsMTx‐4 reduced Ca^2+^ influx into the cells treated with Yoda1.^[^ ^23^ ^]^

**Figure 4:**
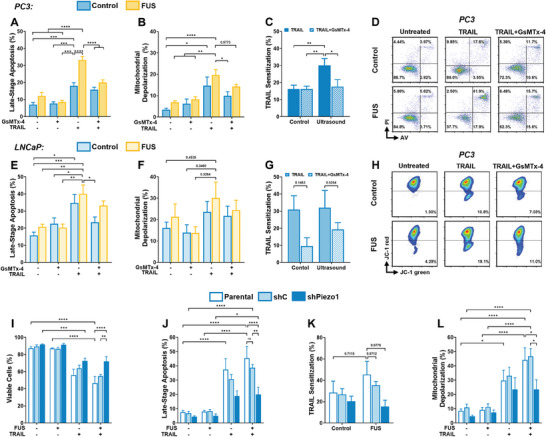
Piezo1 inhibition and knockdown reduces the effects of TRAIL sensitization. PC3 and LNCaP cells were exposed to 60 s of ultrasound at 944 kPa and treated with GsMTx‐4, TRAIL and ultrasound at 944 kPa for 60 s. PC3 summary data showing the mean percentage of A) late‐stage apoptosis, B) mitochondrial depolarization, and C) TRAIL sensitization (Equations (2)–(4)). D) Representative AV/PI flow cytometry plots for the PC3 cells. LNCaP summary data showing the mean percentage of E) late‐stage apoptosis, (F) mitochondrial depolarization, and G) TRAIL sensitization (Equations (2)–(4)). H) Representative JC‐1 flow cytometry plots showing the PC3 cells. Summary data for the knockdown of *PIEZO1* in the PC3 cells showing the mean percentage of I) viable cells, J) late‐stage apoptosis, K) TRAIL sensitization (Equations (2) and (3)), and L) mitochondrial depolarization for the parental, control (shC) and *PIEZO1* KD (shPiezo1) conditions. *n* = 4 (A–C), *n* = 5 (E–G), and *n* = 3–4 independent experiments (I–L); two‐way ANOVA (A–C, E–G, I–L). *p < 0.05, **p < 0.01, ***p < 0.005, ****p < 0.0001. Error bars represent mean ± SEM.

Pre‐treatment of PC3 and LNCaP cells with GsMTx‐4 prior to FUS+TRAIL treatment showed similar results to Figure S5 (Supporting Information), in which we alternatively used EGTA to chelate Ca^2+^ in the experimental buffer media. A significant reduction in late‐stage apoptosis was seen for the PC3 cells treated with FUS+TRAIL+GsMTx‐4 (**Figure** **4**A,D), decreasing from 33.3 ± 1.90% for the FUS+TRAIL treatment to 20.1 ± 1.48% when GsMTx‐4 was introduced. Additionally, the TRAIL sensitization reduced from 30.15 ± 3.96% for the FUS+TRAIL group to 17.7 ± 4.12% for the FUS+TRAIL+GsMTx‐4 group (Figure 4C). The extent of depolarized mitochondria (Figure 4B,H) showed a significant decrease for the FUS+TRAIL treatment following GsMTx‐4 incubation, consistent with the TRAIL sensitization. These effects were notable, yet less significant in the LNCaP cells. The percentage of LNCaP cells in late‐stage apoptosis decreased 1.21‐fold following GsMTx‐4 treatment when exposed to the FUS+TRAIL therapy (Figure 4E). Similar trends were observed for the mitochondrial depolarization (Figure 4F) and TRAIL sensitization (Figure 4G).

Chelation of Ca^2+^ (Figure S5, Supporting Information) reduced the therapeutic efficacy of the FUS+TRAIL therapy in PC3 and LNCaP cells, as evidenced by the reduced late‐stage apoptosis, mitochondrial depolarization and TRAIL sensitization for the FUS+TRAIL therapy following incubation of EGTA in the experimental media. For instance, the TRAIL sensitization decreased from 32.6 ± 8.12% at the FUS+TRAIL condition to 13.4 ± 5.82% at the FUS+TRAIL+EGTA condition in PC3 cells (Figure S5d, Supporting Information).

To validate these findings, we knocked down the *PIEZO1* gene using shRNA lentiviral particles (shPiezo1) in PC3 cells (Figure 4I–L). Successful knockdown was validated using flow cytometry (Figure S6, Supporting Information). *PIEZO1* knockdown decreased the percentage of cells in late‐stage apoptosis for the FUS+TRAIL treatment compared to the parental and control (shC) conditions (Figure 4J). Additionally, the TRAIL sensitization for the parental and shC conditions treated with FUS resulted in mean values of 45.4 ± 12.3% and 35.4 ± 3.56%, respectively, while the TRAIL sensitization of the shPiezo1 cells exposed to FUS remained at 15.5 ± 5.88% (Figure 4K). The percentage of mitochondrial depolarization in the shPiezo1 cells also remained unchanged following the FUS+TRAIL treatment compared to the TRAIL‐only treatment, and remained significantly lower than the parental and shC conditions (Figure 4L).

Further, we explored the use of 2‐APB (Figure S7, Supporting Information) to inhibit multiple (transient receptor potential) TRP channels simultaneously, including TRPC, TRPV, and TRPM channels, since some TRP channels also regulate Ca^2+^.^[^ ^51^, ^52^ ^]^ We confirmed that any activation of TRP channels by FUS are not significantly contributing to the elevated cell death observed after FUS+TRAIL treatment by testing 2‐APB at a lower (10 µm), medium (50 µm), and higher (100 µm) concentration in PC3 cells. Insignificant differences were observed between the control and FUS groups at all three inhibitor concentrations, consistent with the viability (Figure S7a, Supporting Information), late‐stage apoptosis (Figure S7b, Supporting Information), TRAIL sensitization (Figure S7c, Supporting Information), and mitochondrial depolarization (Figure S8d, Supporting Information). Therefore, we did not test this inhibitor further in the LNCaP cells.

These results exemplify that disruption of the mechanoactivation of Piezo1 affects the efficacy of this combination therapy to induce apoptosis. Importantly, no significant increase in apoptosis was evident for the EGTA, GsMTx‐4 or shPiezo1 treatment only. Altogether, these observations illustrate that Piezo1 is an integral component to mediate the enhanced TRAIL sensitization observed when FUS is applied to mechanically evoke intrinsic tumor cell apoptosis, as pre‐treatment of PC3 cells with 2‐APB showed that TRP activation via FUS is not the primary mechanism driving enhanced apoptosis following FUS+TRAIL therapy.

### 2.4 Apoptosis Array Suggests Synergistic Apoptosis Mechanism

**Figure 5:**
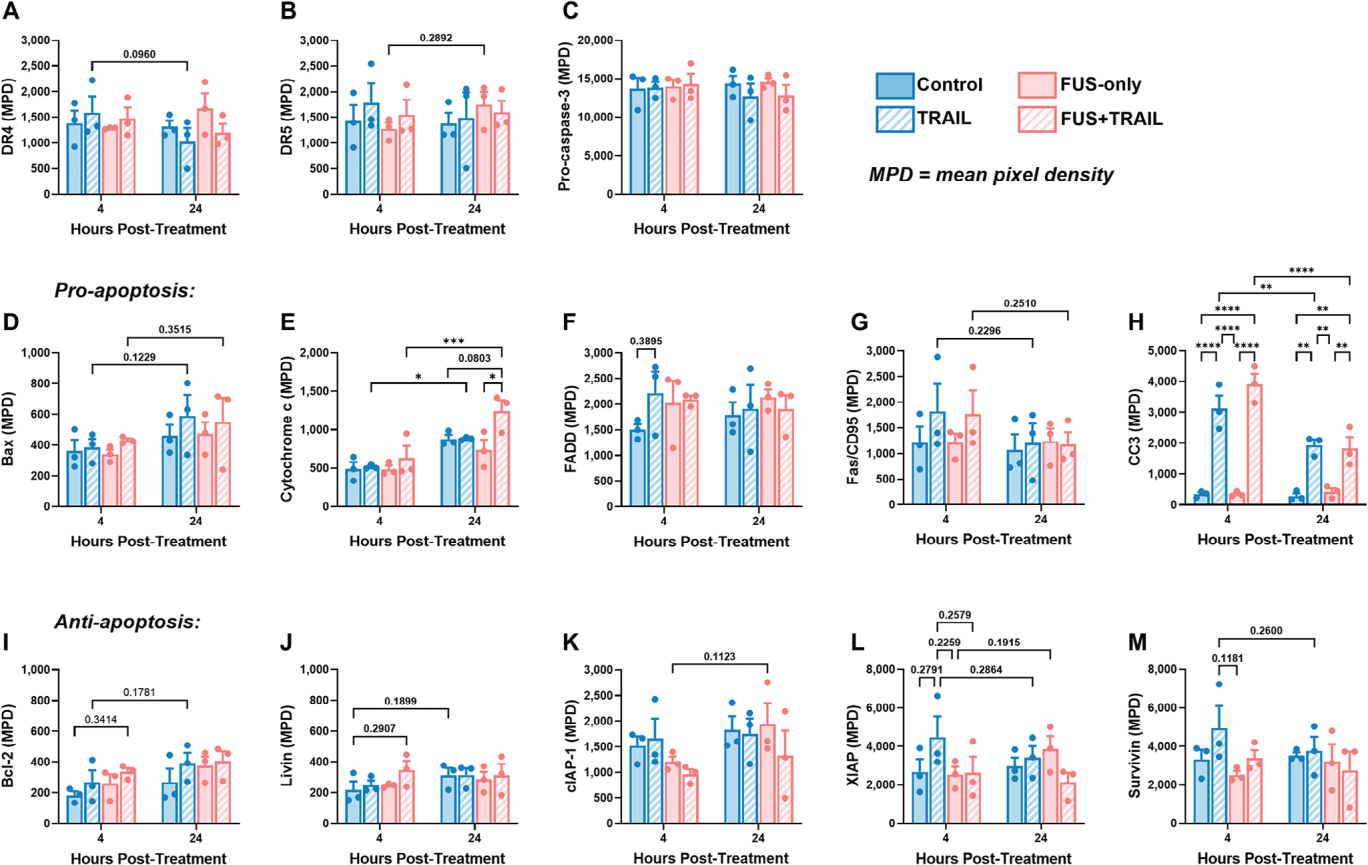
Apoptosis‐related protein expression. Expression of various proteins involved in the apoptotic pathway determined via the apoptosis proteome profiler array for the PC3 cells at 4 and 24 h post‐treatment. Mean pixel density (MPD) of A) DR4, B) DR5, C) pro‐caspase‐3, D) Bax, E) Cytochrome c, F) FADD, G) Fas/CD69, H) cleaved caspase‐3 (CC3), I) Bcl‐2, J) Livin, (K) cIAP‐1, (L) XIAP, and (M) Survivin. *n* = 3 independent experiments; two‐way ANOVA. *p < 0.05, **p < 0.01, ***p < 0.005, ****p < 0.0001. Error bars represent mean ± SEM.

To further investigate the mechanism behind the observed TRAIL‐mediated apoptosis, we used a human apoptosis array to examine the expression of various apoptosis‐related proteins at 4 and 24 h post‐treatment in PC3 cells (**Figures** **5** and S8, Supporting Information). Overall, DR4/5 expression levels (Figure 5A,B) did not show significant changes. Cleaved caspase‐3 (CC3) is an executioner caspase that cleaves DNA at specific sites to induce apoptosis and becomes activated once pro‐caspase 3 is cleaved by caspase‐8 and caspase‐9.^[^ ^53^ ^]^ Therefore, CC3 is a marker of apoptosis in the downstream, synergistic TRAIL‐Piezo1 activation pathway.^[^ ^23^, ^46^, ^54^, ^55^ ^]^ We observed a significant increase in CC3 for the TRAIL and FUS+TRAIL‐treated conditions at 4 h (Figure 5H), while the pro‐caspase‐3 expression remains unchanged (Figure 5C). At 4 h, the mean CC3 expression for the TRAIL treatment condition is 3.13 × 10^3^ ± 418 mean pixel density (MPD) and 3.91 × 10^3^ ± 347 MPD for the FUS+TRAIL condition. At 24 h, there is a reduced CC3 expression for the TRAIL conditions. This indicates that the majority of the intrinsic apoptosis occurred prior to the 24 h mark following treatment.

Additionally, cytochrome c is an apoptogenic protein released from the mitochondria into the cytosol during intrinsic apoptosis.^[^ ^12^, ^23^, ^27^, ^56^ ^]^ 4 h post‐treatment, the cytochrome c level is highest for the FUS+TRAIL condition (631 ± 159 MPD) and increases by 1.97‐fold at 24 h (Figure 5E). For all conditions, the cytochrome c level increases 24 h post‐treatment, which could partially be explained as a net increase in protein expression during the longer incubation period. FADD is an important protein involved in DR signaling, containing a death domain that interacts with the death domain of DR4/5. Once FADD is recruited to the DR complex, it can further activate downstream signaling molecules to induce apoptosis.^[^ ^56^ ^]^ FADD expression was highest at 4 h for the TRAIL (2.23 × 10^3^ ± 726 MPD) and FUS+TRAIL (2.10 × 10^3^ ± 123 MPD) conditions (Figure 5F) and decreased slightly at the 24 h timepoint. Fas/CD95 is an apoptosis‐inducing receptor that sits on the cell surface to recruit pro‐apoptotic factors (i.e., caspase‐8) to induce cell death extrinsically.^[^ ^57^ ^]^ Therefore, significant shifts in Fas/CD95 expression are not expected. However, we observed that the TRAIL‐treated conditions demonstrated the highest Fas/CD95 expression at 4 h post‐treatment (Figure 5G).

Expression of the anti‐apoptotic proteins Bcl‐2 and livin remained low overall (Figure 5I,J). Members of the inhibitor of apoptosis (IAP) family, cIAP‐1 and XIAP, were also measured (Figure 5K,L). PC3 cells have been shown to have elevated levels of cIAP‐1 and XIAP.^[^ ^58^ ^]^ For the FUS+TRAIL condition, cIAP‐1 expression remained the lowest for both timepoints, increasing only 1.2‐fold between 4 and 24 h. XIAP directly inhibits caspases‐3, ‐7, and ‐9 and at 24 h, and XIAP expression was the lowest for the FUS+TRAIL conditions (2.13 × 10^3^ ± 483 MPD).^[^ ^59^ ^]^ Lower cIAP‐1 and XIAP expression for the FUS+TRAIL condition is consistent with significantly higher apoptosis for the FUS+TRAIL therapy, further supporting activation of a synergistic TRAIL‐Piezo1 apoptotic pathway. Furthermore, decreased survivin expression for the FUS+TRAIL group 24 h post‐treatment was observed (Figure 5M). Survivin binds to smac during apoptosis to prevent smac/DIABLO from binding to and activating IAPs.^[^ ^60^ ^]^

### 2.5 Multiple FUS+TRAIL Doses Significantly Reduce Tumor Burden In Vivo

**Figure 6:**
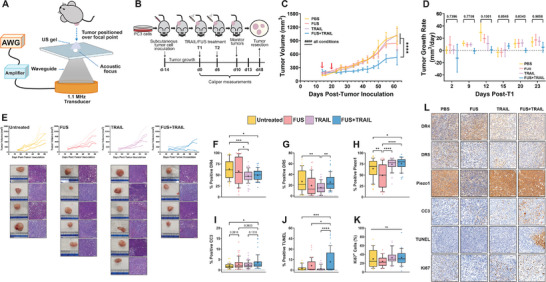
Mechanotherapy reduces tumor burden. A) In vivo ultrasound apparatus. B) Treatment timeline, C) mean tumor volume (Equation (7)), and D) mean tumor growth rate (Equation (8)) for PC3 flank tumors receiving two treatments on day 0 and day 6 post‐tumor inoculation (day −14). Red arrows indicate treatment days. E) Individual tumor curves with tumor photographs (ruler showing cm) and corresponding H&E staining shown below. Percent positive IHC stains for F) DR4, G) DR5, H) Piezo1, I) CC3, J) TUNEL, and K) Ki67 for the tumors following resection. L) Representative IHC micrographs (scale bar = 100 µm). *n* = 5–6 tumors per condition; two‐way ANOVA (C, D) and one‐way ANOVA (F–K). *p < 0.05, **p < 0.01, ***p < 0.005, ****p < 0.0001. Simple linear regression to confirm significant deviation from zero (C), ####*p* < 0.0001. Error bars represent mean ± SEM (C, D). Box and whisker plots show individual data points outside of the 10–90th percentile with mean indicated as (+), median indicated as the solid horizontal line and outliers indicated as individual points (F–K).

Few studies have investigated the intratumoral administration of recombinant, soluble, human TRAIL in vivo.^[^ ^61^, ^62^, ^63^ ^]^ A single treatment in vivo pilot study (data not shown) motivated further testing of the FUS+TRAIL therapy on a larger scale, creating the potential for greater differences in tumor burden. Thus, we sought to investigate the effects of two FUS+TRAIL treatments (T1 and T2) administered six days apart, to discern the long‐term effects on tumor growth until endpoint. Phantom simulations and measurements were completed to confirm no major thermal effects from the FUS in vivo, and we observed a maximum temperature rise of 2.43 °C after 10 min of sonication was carried out (Figure S9, Supporting Information). PC3 flank tumors were inoculated on the flanks of male, NU/NU mice at day −14 (**Figure** **6**). A single dose of TRAIL at 2 mg kg^−1^ was administered, and a single FUS exposure was defined as 10 min of sonication, with a PRF of 0.100 Hz, pressure of 944 kPa and pulse duration of 500 ms. The in vivo FUS apparatus is shown in Figure 6A and the tumors were positioned over the focal point of the transducer coupled with ultrasound gel. The treatments were given on day 0 and day 6, two weeks following tumor cell inoculation (Figure 6B).

Tumor volume was measured for 62 days following tumor cell inoculation (Figure 6C,E). The tumor burden for the FUS+TRAIL‐treated tumors was significantly lower for the duration of the study, compared to the PBS or single‐treatment tumors. On day 62 post‐tumor cell inoculation, the mean volume of the FUS+TRAIL tumors was 529 ± 170 mm^3^. PC3 tumors were confirmed using hematoxylin and eosin (H&E) staining (Figure 6E). The tumor growth rate for ≈2 weeks post‐T1 (Figure 6D) was lowest for the FUS+TRAIL condition showing an initially slowed tumor progression, except for day 15 post‐T1 where the growth rate reached 7.20 ± 4.16 mm^3^ day^−1^, which is 9 days after T2. These results suggest the short half‐life of soluble TRAIL in circulation is irrelevant in the vastly different and compact tumor environment.

Tumors were processed for immunohistochemistry (IHC) staining (Figure 6F–L) and analyzed by measuring the percentage of positive expression of several markers involved in the initiation and execution of the intrinsic apoptotic pathway. Since the tumors were resected and fixed 39 days after T2, the significant variations in some of the IHC results are likely due to regrowth of PC3 cells in regions of the tumors receiving treatments that induced apoptosis. FUS or TRAIL treatment was not expected to alter DR4/5 expression. Considering that the mean DR4 expression is lowest for the TRAIL (47.0%) and combination‐treated tumors (49.6%), this could be due to DR4 receptor internalization once TRAIL has become bound to its receptor on the cancer cells (Figure 6F).^[^ ^64^ ^]^ These results are consistent with the 24 h timepoint in Figure 5A as determined by the apoptosis profiler. We confirmed that most cells express DR4/5 when they are fixed without permeabilization, compared to fixation and permeabilization (Figure S10, Supporting Information). Therefore, it is likely that the DR4/5 antibodies used for IHC staining are primarily binding to the extracellular DR4/5 receptors. Additionally, mice only express DR5, therefore DR4+ regions also confirm the presence of human PC3 tumor cells.^[^ ^65^ ^]^ DR5 expression for the combination treatment (25.6%) was similar to the PBS treatment (27.0%), and slightly higher compared to the FUS‐ or TRAIL‐only treatments (Figure 6G), consistent with the single treatment pilot study we performed (not shown). These results indicate that the administration of TRAIL followed directly by FUS does not affect the expression of DR5 in PC3 tumors. Overall, the DR4 expression remained higher than the DR5 expression for all treatments compared, consistent with in vitro results captured via confocal imaging (Figure S11, Supporting Information).^[^ ^66^ ^]^

*PIEZO1* gene expression is shown to increase in cancerous prostate tissue (Figure S12, Supporting Information), and increased Piezo1 protein expression promotes PCa progression.^[^ ^67^, ^68^, ^69^ ^]^ The Piezo1 expression was lowest for the FUS treatment (49.2%) (Figure 6H). Interestingly, there is an increase in Piezo1 expression for the combination treatment compared to the FUS‐ and TRAIL‐only treatments. For FUS alone, the Piezo1 expression was found to decrease. We cannot assume that changes in Piezo1 expression are directly associated with alterations in Piezo1 activation, since this relationship is not well understood. However, increased Piezo1 expression could also be a mechanoresponse to inflammation.^[^ ^70^ ^]^

Overall, the percentage of CC3 remained low for all conditions, and was 1.8‐fold higher for the FUS+TRAIL‐treatments compared to the PBS condition (Figure 6I). Terminal deoxynucleotidyl transferase‐mediated dUTP‐nick end‐labeling (TUNEL) is a marker of the last phase of apoptosis and necrosis that detects breaks in DNA when fragmentation occurs.^[^ ^71^, ^72^ ^]^ Successful binding of TRAIL to DR4 is expected to be consistent with the enhanced positive expression of CC3 (Figure 6I) and TUNEL (Figure 6J) for the FUS+TRAIL treatment compared to all other groups. This could indicate that the FUS as an added therapy enhanced these effects. The FUS‐only treated tumors also showed higher TUNEL expression compared to the PBS and TRAIL‐treated tumors (Figure 6J). Therefore, the 10 min FUS exposure alone might contribute to some of the cell death observed in vivo since the transducer is directly focused on the tumors. The mean TUNEL expression for the FUS+TRAIL‐treated group was 1.8‐fold higher than the FUS‐only‐treated tumors, 4.7‐fold higher than the PBS‐treated tumors, and 5.7‐fold higher than the TRAIL‐treated tumors. Differences in expression between CC3 and TUNEL is likely because TUNEL indicates both apoptosis and necrosis.^[^ ^71^, ^72^ ^]^ The proliferation within the tumors showed no significant variation among the different treatment groups (Figure 6K).

### 2.6 Analysis of Treatment Intervals of Mechanotherapy In Vivo

**Figure 7:**
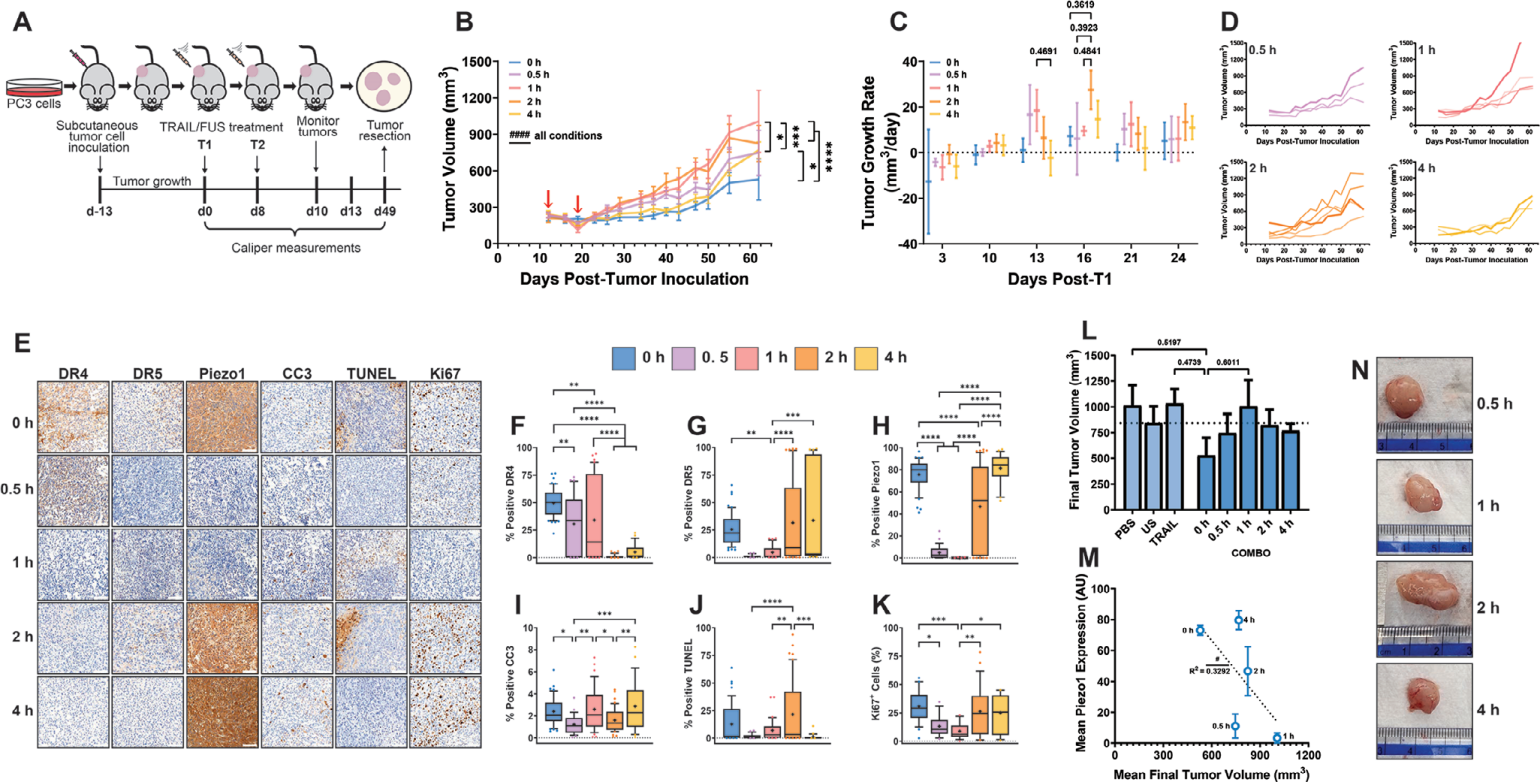
Effects of varied timepoints between treatments. A) Treatment timeline, B) mean tumor volume (Equation (7)), and C) mean tumor growth rate (Equation (8)) for PC3 flank tumors receiving two treatments on day 0 and day 8 post‐tumor inoculation (day −13). Red arrows indicate treatment days. D) Individual tumor curves. E) Representative IHC micrographs (scale bar = 100 µm). Percent positive IHC stains for F) DR4, G) DR5, H) Piezo1, I) CC3, J) TUNEL, and K) Ki67 for the tumors following resection. L) Comparison of final mean tumor volumes for tumors in (B) and (Figure 6C). M) Correlation between mean Piezo1 expression and mean final tumor volume with best fit line. N) Representative tumor images with ruler showing cm. The 0 h treatment group is the same as the FUS+TRAIL treatment group in Figure 6, shown for comparison. *n* = 3–5 tumors per condition; two‐way ANOVA (B, C, E) and one‐way ANOVA (F–K). *p < 0.05, **p < 0.01, ***p < 0.005, ****p < 0.0001. Linear regression to confirm significant deviation from zero (B, M), #*p* < 0.05, ####*p* < 0.0001. Error bars represent mean ± SEM (B, C, L, M). Box and whisker plots show individual data points outside of the 10–90th percentile with mean indicated as (+), median indicated as the solid horizontal line and outliers indicated as individual points (F–K).

We were also interested in performing a temporal treatment regimen, in which various time intervals between the TRAIL injection and FUS exposure were studied: 0, 0.5, 1, 2, and 4 h. The same FUS and TRAIL parameters were employed as in Figure 6. Treatments were given on day 0 and day 8 (**Figure** **7**A) and H&E staining of tumors upon resection confirmed PC3 cells (Figure S13, Supporting Information). Representative tumor images are shown in Figure 7N. Figure 7B shows that the tumor burden was lowest for the 0 h treatment group, which is the same as the FUS+TRAIL tumor group used in Figure 6C. This was followed closely by the 4 h treatment group. Overall, the 1 and 2 h intervals permitted the highest tumor burden for the duration of the study. The 4 h timepoint responded similarly to the 0 h timepoint, which could imply that the TRAIL binding either occurs more quickly in the presence of FUS, or that without an additional stimulus, TRAIL may require a prolonged period to diffuse and successfully recognize and bind to the individual cells within the compact tumor environment.^[^ ^30^, ^35^ ^]^

Comparing the final tumor volume of the treatment types (Figure 6C) to the treatment intervals (Figure 7B) shows that the 0, 0.5, 2, and 4 h treatment intervals had a lower mean tumor volume on the day of tumor resection compared to the PBS, FUS, and TRAIL‐treated tumors (Figure 7L). The final mean tumor volume for the 1 h treatment interval (1006 ± 255 mm^3^) was comparable to the PBS (1014 ± 198 mm^3^) and TRAIL (1032 ± 143 mm^3^) treatments. The tumor growth rate for ≈2 weeks post‐T1 was the lowest for the 0 h interval for most days (Figure 7C). Three days after T2 (day 13 post‐T1), the 0.5 and 1 h intervals show a higher growth rate of 16.6 ± 13.0 and 18.4 ± 9.12 mm^3^ day^−1^, respectively. Six days after T2 (day 16 post‐T1), the growth rate was enhanced for the 2 h interval (27.5 ± 8.53 mm^3^ day^−1^) compared to all other conditions.

The mean DR4 expression was higher for the 0, 0.5, and 1 h treatment intervals compared to the DR5 expression in these treatment groups (Figure 7F,G). The mean DR5 expression was highest for the 0 h treatment interval. These significant variations in DR4/5 expression for similar treatment regimens could also be explained by differences in the receptor activity following TRAIL binding.^[^ ^64^ ^]^ Research has also shown that DR5 promotes cancer cell invasion and metastasis, so increases in DR5 expression and proliferation in response to some of the treatment intervals could indicate a fraction of tumor cells re‐populating in regions of the tumor that previously underwent apoptosis from the treatment, since tumors were resected 41 days after the second treatment and no additional doses were administered.^[^ ^73^, ^74^ ^]^

Correlating with the smallest mean tumor volume, the mean Piezo1 expression (Figure 7H) was highest for the 0 and 4 h treatment intervals, which were 75.6% and 81.5%, respectively. The 0.5 and 1 h treatment intervals showed a mean Piezo1 expression of 4.80% for 0.5 h and 0.12% for 1 h. The 0.5, 1, and 2 h treatment intervals showed larger tumor size compared to the 0 and 4 h treatments, so it is possible that an unknown mechanism following treatment in some tumors promoted Piezo1 downregulation. Figure 6M shows that there is no significant correlation between the mean Piezo1 expression and the mean tumor volume; however, the trend suggests an inverse relationship. This is consistent with the expectation that the conditions that showed significantly lower Piezo1 expression resulted in larger tumor volumes, similar to PBS‐ and FUS‐treated groups in Figure 6.

The mean CC3 expression was highest for the 0 (2.40%), 1 (2.60%), and 4 h (2.87%) treatment intervals (Figure 7I). Increased CC3 in the 1 h treatment interval associated with a higher tumor volume compared to the other treatments could be due to successful activation of caspase‐3, but an incomplete execution of apoptosis.^[^ ^75^ ^]^ The TUNEL expression was significantly higher in the 2 h timepoint (21.5%) (Figure 7J), which is noteworthy since this tumor treatment interval had one of the higher tumor volumes and an increase in proliferation (Figure 7K). It is possible that the tumors receiving 2 h treatment intervals happened to be more heterogeneous, in which these tumors expressed more regions of dead cells, despite maintaining larger tumors overall. Proliferation was highest for the 0 (30.8%), 2 (26.3%), and 4 h (25.1%) intervals (Figure 7K). Although the mice used in these studies were athymic and unable to produce T cells, there could still be a minimally enhanced immune cell response or infiltration within the tumors post‐treatment, involving subpopulations such as B‐cells or dendritic cells.^[^ ^76^ ^]^ FUS has been shown to enhance proliferation in fibroblasts and inhibit proliferation in LNCaP and PC3 cells.^[^ ^77^, ^78^ ^]^

### 2.7 Combination Therapy Does Not Promote EMT Phenotype

We also examined if there were any changes in expression of EMT‐associated proteins, E‐cadherin, vimentin, and pan‐cytokeratin (CK) (Figure S14, Supporting Information). Generally, an increased vimentin expression and decreased E‐cadherin and CK expression signify EMT progression and enhanced migratory behavior in cancer cells.^[^ ^79^ ^]^ The vimentin expression remained similar across all treatments (Figure S14a, Supporting Information) and is highly expressed in PC3 cells due to their highly metastatic phenotype.^[^ ^37^ ^]^ Vimentin expression was highest for the 0 h treatment group (81.5%), decreasing most notably for the 0.5 and 1 h regimens (Figure S14d, Supporting Information). LIFU has been shown to decrease vimentin expression, reversing the EMT process which could be indicative of halting or slowing cancer progression.^[^ ^80^ ^]^

The CK expression was relatively low for the PBS, TRAIL, and combination treatments, but significantly increased for the FUS‐treated tumors (Figure S14b, Supporting Information). The CK expression was highest for the 0 and 4 h treatment intervals for the combination therapy (Figure S14e, Supporting Information), which is consistent with the decreased tumor burden for these conditions. A significant increase in E‐cadherin expression was observed for the FUS+TRAIL treatment group (0.06%) compared to the control groups (Figure S14c, Supporting Information), and overall PC3 cells express very little E‐cadherin. Insignificant variation was observed among the different treatment intervals (Figure S14f, Supporting Information); however, these values all remained equal to or greater than 0.05%, higher than the expression levels for the individual treatments in Figure S14c (Supporting Information). One may conclude that this therapy does not significantly promote changes in EMT phenotype in PCa cells.

## 3 Discussion

Development of noninvasive, safe, and effective treatment options for PCa are sorely needed, considering the risk that other therapeutic options impose on the patient's quality of life. The mechanotherapy presented here exemplifies how FUS can target tumors and enhance the pro‐apoptotic effects of soluble TRAIL.^[^ ^81^ ^]^ The maximum pressure examined was 1993 kPa, and most experiments were performed at or below 944 kPa, at which the mechanical index is 0.9. An MI of 1.9 is the limit considered safe by the FDA before any major cavitation is expected to occur; higher values of MI increase the probability of mechanical damage.^[^ ^39^, ^40^, ^42^ ^]^ Mechanical index is calculated as the peak negative pressure in MPa divided by the square root of the frequency in MHz. For the in vitro experiments, minimal cell death was observed from FUS treatment alone in comparison to 0 kPa or control groups without TRAIL. The initial studies which examined the response of the LNCaP and PC3 cell lines to this combination therapy elucidated the fact that this therapy would need to be modified to yield more successful results for cell lines more resistant to TRAIL, yet showed success in ablating a highly metastatic cancer cell line. Increasing the FUS pressure for a 60 s sonication exposure for both cell lines showed an increase in late‐stage apoptosis and mitochondrial depolarization for the TRAIL‐treated conditions (Figure 2). These results confirmed that this apoptosis was executed via the intrinsic apoptosis pathway.^[^ ^12^, ^23^ ^]^ The experiments in Figures 3, 4, 5, 6, 7 that followed were conducted using primarily the PC3 cells since this cell line represents advanced disease, providing greater clinical relevance. We continued to refine the in vitro FUS parameters by confirming that doubling the FUS exposure time for the PC3 cells had no significant effect on the TRAIL‐mediated apoptosis or mitochondrial depolarization at a low FUS pressure (380 kPa), indicating that 60 s of FUS was sufficient to induce an effective mechanotherapy (Figures S1 and S2, Supporting Information). At 60 s of FUS, we showed that there was a direct, significant correlation between the FUS pressure and the extent of late‐stage apoptosis, TRAIL sensitization and mitochondrial depolarization (Figure 3).

To confirm our hypothesis that the TRAIL‐mediated apoptosis occurs through synergistic Piezo1 activation by FUS, we employed the use of EGTA and GsMTx‐4 to chelate Ca^2+^ and inhibit Piezo1, respectively. We performed these experiments at 944 kPa. Pre‐incubation of our samples with either of these constituents reduced TRAIL‐mediated apoptosis (Figures 4 and S5, Supporting Information). The effects of GsMTx‐4 and EGTA were less pronounced in the LNCaP cells compared to PC3 cells owing to the differential MSC gating dynamics and physiological origins of these cell lines. For instance, Maroto et al. have shown that LNCaP cells display a rapidly inactivating and mechanically fragile Ca^2+^‐permeable MSC current, whereas PC3 cells present a sustained current that remains open during mechanical stimulation.^[^ ^82^ ^]^ This may explain in part why the observed apoptosis of LNCaP cells treated with FUS+TRAIL+GsMTx‐4 was not reduced as significantly (Figure 4E). Additionally, PC3 cells are known to be highly metastatic compared to LNCaP cells, and Piezo1 expression is directly correlated with aggressiveness contributing to cell function such as proliferation, migration and invasion in PCa.^[^ ^47^, ^68^, ^69^ ^]^ Elevated apoptosis and mitochondrial depolarization (Figure S5, Supporting Information) were observed for the TRAIL+EGTA‐treated LNCaPs likely because these cells are an epithelial‐derived cell type, and therefore rely more heavily on Ca^2+^ to prevent cell death and maintain cytoskeletal stability, allowing them to withhold elevated sensitivity to TRAIL.^[^ ^46^, ^83^ ^]^ Thus, chelating Ca^2+^ on its own is expected to affect the viability of these cells by disrupting the cellular homeostasis, inducing endoplasmic reticulum stress, and cause imbalances with the release of pro‐apoptosis factors (i.e., cytochrome c), leading to cell cycle arrest.^[^ ^12^, ^46^, ^84^, ^85^, ^86^ ^]^ Ca^2+^ ions may also influence the expression and clustering of DR4/5 on the cell surface, and this phenomena is understudied between different cell types.^[^ ^72^ ^]^

GsMTx‐4 can inhibit channels from the Piezo and TRP families, but has shown to be most effective at inhibiting Piezo1.^[^ ^12^, ^23^, ^49^, ^87^ ^]^ Our group has previously used both Yoda1 and shear stress to activate Piezo1, and showed that either of these agonists combined with TRAIL treatment is just as effective to synergistically induce apoptosis in comparison to FUS employed here.^[^ ^12^, ^23^ ^]^ Several TRP channels have been shown to be activated by mechanical stimuli, such as TRPV5 and TRPV6.^[^ ^88^, ^89^ ^]^ Specifically inhibiting TRP channels in PCa cells using 2‐APB with the FUS and TRAIL conditions held constant (Figure S7, Supporting Information) further supports our hypothesis that Piezo1 is driving the enhanced TRAIL sensitization. No significant differences in cell death or mitochondrial depolarization were observed for the cells that received combination treatments versus those that did not, even at three different 2‐APB concentrations. Unlike Piezo1, expression of some of the TRP channels, such as TRPC1 and TRPM8, decrease with the progression of PCa and are not as prognostic in the diagnosis of disease.^[^ ^51^, ^90^, ^91^ ^]^ We continued these mechanistic studies and knocked down *PIEZO1* in PC3 cells to further confirm our results (Figure 4I–L), showing less apoptosis for FUS+TRAIL treatment compared to the control groups. Altogether, these results indicate that Piezo1 predominantly mediates the observed synergistic apoptosis.

The apoptosis array results show that CC3 expression was most significantly affected by the TRAIL and FUS+TRAIL treatments compared to the control and FUS‐only conditions (Figure 5H). CC3 can be indicative of extrinsic and intrinsic apoptosis mechanisms. The elevated CC3 expression for the TRAIL‐only condition may be attributed to execution of the extrinsic pathway.^[^ ^92^, ^93^ ^]^ More pronounced changes in Bax expression (Figure 5D) were likely not detected since the apoptosis array measured total Bax protein levels in the cell lysates, whereas active Bax undergoes a specific isoform change that cannot be distinguished independently using this assay. However, previous research in our lab has shown that active Bax expression levels in PC3 cells increased following Yoda1+TRAIL treatment compared to TRAIL treatment alone, so one would expect to see a similar shift.^[^ ^12^ ^]^ Insignificant trends were observed between FADD and Fas/CD95 expression (Figure 5F,G). Although FADD is necessary to form the death‐inducing signaling complex (DISC) to trigger apoptosis upon DR4/5 recognition and binding of TRAIL, one does not expect to see changes in the overall protein expression levels of FADD.^[^ ^56^, ^94^ ^]^ However, researchers have speculated that FADD expression is directly correlated to DR expression.^[^ ^95^ ^]^ Cytochrome c expression was still quite low at 4 h for all conditions, including the FUS+TRAIL group. Significantly higher cytochrome c levels were observed for the combination therapy at 24 h, which could be due to increased synthesis of cytochrome c, in addition to, and in response to, redistribution from the mitochondria to the cytosol.^[^ ^96^, ^97^ ^]^ If one were to knockdown or knockout the cytochrome c gene (*CYCS*) in PC3 cells, one would expect to see an even more dramatic decrease in TRAIL sensitization upon treatment with FUS+TRAIL, based on previous studies conducted by our lab.^[^ ^12^, ^27^ ^]^ In future experiments, it would be interesting to measure caspase‐9 expression since cytochrome c directly activates caspase‐9 in the intrinsic apoptosis pathway.^[^ ^23^, ^56^ ^]^

Decreased cIAP‐1 and XIAP expression for the FUS+TRAIL condition is consistent with significantly increased apoptosis (Figure 5K,L). Thus, it is possible that the combined FUS+TRAIL treatment induces a decrease in cIAP‐1 or XIAP expression that assists in driving the synergistic pathway to execution. These results also indicate that XIAP and survivin (Figure 5L,M) may play a larger role than is fully recognized in the TRAIL‐Piezo1 synergistic apoptosis compared to many of the other apoptosis‐related proteins examined, due to their high baseline expression levels and the larger decrease observed for the FUS+TRAIL treatment at 24 h. It is also likely that the increase in Bax expression at 24 h may contribute to driving a decrease in survivin expression via induction of mitochondrial depolarization.^[^ ^23^, ^54^ ^]^ Additionally, the loss of cIAP‐1, cIAP‐2, and XIAP has been shown to sensitize cancer cells to apoptosis, and treatment of PCa with therapeutic regimens such as resveratrol has also been shown to downregulate cIAP‐1 expression, enhancing DR‐mediated apoptosis.^[^ ^58^, ^98^ ^]^ Treatment of cancer cells with nemadipine‐A combined with TRAIL reduced the expression of survivin, increasing TRAIL sensitization.^[^ ^99^ ^]^ For the combination therapy, elevated expression of pro‐apoptotic proteins cytochrome c and CC3, combined with decreased levels of anti‐apoptotic proteins cIAP‐1 and XIAP, support the idea that the synergistic TRAIL‐Piezo1 downstream pathway drives the TRAIL‐mediated apoptosis in PC3 cells.

Our in vivo results (Figures 6 and 7) are consistent with previous reports, which showed that TRAIL treatment on its own failed to reduce tumor size in mice with preclinical xenografts.^[^ ^62^, ^63^ ^]^ All treatment intervals, except for the 1 h timepoint, between the TRAIL injection and FUS exposure for the multi‐dose in vivo studies in Figures 6 and 7 showed smaller tumor volume compared to the PBS, FUS, and TRAIL conditions. Intratumoral transport of small molecules (MW < 1 kDa) occurs by diffusion, however, recombinant human TRAIL is 19.6 kDa. Therefore, the transport of TRAIL throughout the tumor is expected to be slow and it is unlikely that the drug will be fully cleared from the tumor during the treatment timeline of this study.^[^ ^30^, ^35^ ^]^ Antibody drug conjugates injected intratumorally have been shown to have a half‐life of 27 days, and these molecules are generally larger in size than TRAIL.^[^ ^100^, ^101^ ^]^ Specifically, the 0 and 4 h treatment intervals were shown to be most effective for reducing PCa tumor burden (Figure 7B) and slowing tumor growth (Figure 7C). This could also explain why the TRAIL‐only treatment was less effective compared to the FUS‐only treatment in vivo, whereas in vitro the TRAIL‐only treatment proved to be more effective than the FUS‐only treatment. In vitro, the cancer cells were exposed to FUS while suspended in a microcentrifuge tube, where the cells had room to spread out and were not as “tightly packed” as they would be in a tumor environment. Based on the results of this study, we can conclude that the FUS+TRAIL treatment had no adverse effects on healthy, surrounding tissues. The palpable tumors on the mice showed no lesions and the tumor sections that were analyzed showed no evidence of significant thermal ablation or damage, besides regions of necrosis or apoptosis in the cancer cells induced by the therapy. Enhanced CC3 and TUNEL staining was observed for the 0 h combination therapy in Figure 6, indicative of enhanced therapeutic efficacy, consistent with the significantly reduced tumor burden (Figure 6C).

The DR4/5 expression showed more consistent expression levels for the treatment conditions in the multi‐dose study (Figure 6) compared to the varied treatment intervals studied (Figure 7). It is unknown how long TRAIL can remain active and available to bind in the solid tumor environment, thus it is possible that some TRAIL ligands may have continued to be internalized by the tumor cells for days following treatments. Since we confirmed that the DR4/5 antibodies are primarily binding to the extracellular receptors, we can conclude that trends could be associated with DR4/5 receptor internalization upon TRAIL recognition. The apoptosis array results in Figure 5 show that the protein expression levels of DR4/5 are quite similar overall, between treatment types and receptors. Differences measured between the DR4/5 expression in the apoptosis array and confocal imaging (Figure S11, Supporting Information) in vitro are likely due to differences in the techniques used to quantify protein expression, since the apoptosis array captures total protein expression from cell lysate. Previous research has shown that DR5 is internalized independently of DR4, and DR5 has been shown to be the higher affinity receptor at 37 °C.^[^ ^64^, ^102^ ^]^ Following internalization and removal of TRAIL from media, DR4/5 receptors were shown to slowly return to the plasma membrane and were fully restored within 6 h for TRAIL‐sensitive cells.^[^ ^64^ ^]^ With respect to these results, this could indicate that DR4 was available for TRAIL to bind to for up to ≈1 h, until most of the receptors were internalized from previous successful binding events. Considering that a dramatic decrease in DR5 expression is noted 0.5 h after TRAIL injection, this could indicate that within the first 30 min, TRAIL binds to the majority of DR5 exposed on the surface of the tumor cells. Previous studies inhibiting enhancer of zest homolog 2 (EZH2), which promotes transcriptional silencing downstream, led to elevated expression of DR4 in PCa cell lines, and high levels of NF‐kB in tumor cells have been shown to increase DR4/5 expression.^[^ ^61^, ^103^, ^104^ ^]^ Additionally, Jun N‐terminal kinase activation due to endoplasmic reticulum stress was previously shown to upregulate DR4/5 expression.^[^ ^27^ ^]^ These findings support the idea that there could be a downstream, yet unidentified mechanism occurring in response to combination treatment that induces an increase in DR5 expression. Future experiments could delineate additional mechanoresponses that are induced following combination treatment, perhaps complemented by a full computational model of drug diffusion in that setting to overcome this limitation.

Overall, higher Piezo1 expression correlated with lower tumor burden in vivo, and decreased for the FUS condition (Figure 6C,H). Considering that Piezo1 is elevated in PCa, this could indicate that the mechanical effects of FUS alone can both activate and potentially downregulate expression of Piezo1 over time, which would require further studies to fully explore since there is a general lack in knowledge regarding how Piezo1 expression is affected by mechanical forces.^[^ ^105^ ^]^ Piezo1 expression remained quite high for most treatment regimens, and dramatically decreased for the 0.5 and 1 h treatment intervals (Figure 7H). The explanation for this last observation is not yet clear, but could be attributable to heterogeneity between the tumors (i.e., tumors in these groups happen to express less Piezo1 than other treatment groups, not attributable to the FUS or TRAIL treatments), or as part of an inflammatory response that is involved with mechanotransduction responses beyond what has been investigated here.^[^ ^70^ ^]^

We also confirmed that significant changes in tumor size are uncorrelated with changes in EMT phenotype of the PCa cells (Figure S14, Supporting Information), in accordance with previous reports that suggest combination therapies involving FUS reverse the EMT process. This is critical to slow cancer progression.^[^ ^33^ ^]^ Developing a better understanding of how EMT is affected by FUS will yield more insights into how the TME as a whole is affected by FUS+TRAIL therapy. Additionally, research has shown that exposure of mouse 4T1 breast cancer tumors to FUS showed no differences in hemorrhage compared to control tumors.^[^ ^106^ ^]^ However, further exploring ways that FUS affects extracellular matrix composition within the TME would help researchers understand how factors such as collagen deposition mitigate the ability of cancer cells to migrate into the primary TME to metastasize.^[^ ^107^, ^108^ ^]^ The effects of this therapy on the TME can be further studied by performing these in vivo studies on immunocompetent mice to investigate the effects on immune cell infiltration.^[^ ^106^, ^109^ ^]^ Additionally, this would yield understanding of how one may safely couple this combination treatment with additional anti‐cancer therapies working to slow or prevent invasive progression in cancer cells.

The in vivo studies show promising potential for this mechanotherapy to yield successful anti‐cancer results, by significantly decreasing tumor burden noninvasively through only two doses of treatments. To address the limitations of these studies, several modifications can be implemented. The treatments could be altered to further refine the most effective therapeutic approach, such as adding additional FUS exposure times since we determined that the TRAIL is not immediately cleared out of the tumors. Adding additional FUS doses could help prevent re‐proliferation of the tumor cells after a degree of apoptosis from the initial treatments. This would also help the TRAIL penetrate because with attenuated proliferation, the tumor would become less dense.^[^ ^30^ ^]^ The proposed combination therapy may also be used with magnetic resonance or other noninvasive, precise imaging modalities, since these technologies are widely available today to better track and confirm successful injection of the TRAIL and targeting of the FUS.^[^ ^110^ ^]^ Furthermore, microbubbles or other acoustically active molecules could be used in combination with TRAIL to improve drug delivery or allow for lower pressures to be used.^[^ ^111^ ^]^ Future work could introduce local delivery of immune‐stimulating agents or electron‐rich reactive oxygen biocatalysts to further prime the local tumor environment and generate a systemic response, thereby opening up new opportunities to amplify immune checkpoint blockade responses.^[^ ^22^, ^112^, ^113^ ^]^

Combining FUS+TRAIL treatment with US‐contrast agents that are responsive to FUS stimulation could also be implemented to treat CTCs by introducing these agents into liposomal delivery vehicles tagged with TRAIL, as previously developed within our lab.^[^ ^28^ ^]^ Moreover, combination with other therapeutic moieties such as dual affinity nanoparticles that are functionalized with TRAIL or chemotherapies, such as cisplatin, to overcome drug resistance could prove useful.^[^ ^24^, ^25^, ^26^, ^27^, ^28^, ^114^ ^]^ Additional drugs shown previously by our lab to act as TRAIL sensitizers may also be combined with this technology, such as piperlongumine, low dosage aspirin, curcumin, and low dosages of chemotherapies such as docetaxel.^[^ ^27^, ^115^, ^116^, ^117^, ^118^ ^]^ The multi‐modal therapy could also be combined with an additional treatment that targets specific genes that promote cancer cell invasion and migration, such as PCGME1 or BACH1.^[^ ^119^, ^120^ ^]^

FUS+TRAIL treatment can be easily translated to examine the success in treating other cancers. For instance, one of the primary challenges when treating patients with glioblastoma (GBM) is for drugs or chemotherapeutics to penetrate across the blood brain barrier. We determined that the FUS parameters utilized in the present study are safe, do not cause unwanted thermal ablation, and are unlikely to cause damage to bone. The results here also suggest the potential to employ FUS+TRAIL in combination with other therapeutic regimens, such as Temozolomide, to enhance the anti‐cancer effects to treat GBM to create a three‐component therapeutic approach.^[^ ^23^, ^121^ ^]^

LIFU is in an earlier stage of development; the clinical doses are not as well understood compared to HIFU and limitations still exist. Some tumors may be inaccessibly reached by the FUS beam, however this may be overcome through the use of an intracavity transducer.^[^ ^122^ ^]^ Nonetheless, many benefits support the adoption of low‐intensity FUS to treat cancer. Low‐intensity FUS can target superficial and deep brain structures, with less risk of bone fracture that HIFU may contribute to under certain conditions.^[^ ^123^, ^124^ ^]^ Additionally, there does not seem to be any limit to the number of treatments a patient can receive when used for pain management, and FUS treatment tends to be more affordable than other treatments.^[^ ^123^, ^125^ ^]^ Within this study, our efforts have focused on examining the success of this therapy to treat solid tumors. However, future work will be needed to further optimize FUS operating parameters. Ultrasound parameters are extremely customizable and the effects of pulse length, pulse repetition frequency, treatment duration and duty cycle warrant further research for the optimization of low‐intensity modulation.^[^ ^126^, ^127^ ^]^

## 4 Conclusion

In conclusion, this work prevents a safe, noninvasive, and highly translatable mechanotherapy to treat cancer, which is based on the synergistic activation of Piezo1. The experiments presented here were performed at a safe MI, which provides advantages over similar, more intense therapies such as HIFU, by reducing off‐target tissue ablation and avoiding hyperthermia. Enhancing the therapeutic efficacy of local delivery of cancer therapeutics is critical to reduce side effects and prevent formation of metastases.

## 5 Experimental Section

### Cell Culture

Prostate adenocarcinoma cell lines PC3 (ATCC #CRL‐1435) and LNCaP (ATCC #CRL‐1740) were purchased from American Type Culture Collection (Manassas, VA, USA). LNCaP and PC3 cells were cultured in RPMI 1640 cell culture medium, supplemented with 10% (v/v) fetal bovine serum (Gibco), 1% (v/v) PenStrep (Fisher Scientific, Carlsbad, CA, USA), and 10 mm HEPES (v/v) (Fisher Scientific). LNCaP media was also supplemented with 1 mm sodium pyruvate (Fisher Scientific). Cells were incubated under humidified conditions at 37 °C and 5% CO_2_, and did not exceed 90% confluence.

### Cell Preparation for Ultrasound Studies

LNCaP and PC3 cells were washed in HBSS free of Ca^2+^ and Mg^2+^ (HBSS) (Corning, Manassas, VA, USA), followed by treatment with 0.25% trypsin‐EDTA (Gibco) for PC3 cells or Accutase (Sigma‐Aldrich, St. Louis, USA) for LNCaP cells for 5–6 min at 37 °C. Cells were spun at 300×*g* to remove dissociation agent and resuspended in complete media at a concentration of 0.2 × 10^6^ cells mL^−1^ prior to ultrasound studies. 4 × 10^5^ cells were pipetted into 2 mL microcentrifuge tubes (Corning) to prepare for experimentation.

For studies involving TRAIL, cells were treated with 200 ng mL^−1^ recombinant human TRAIL (PeproTech, Rocky Hill, NJ, USA) immediately before ultrasound exposure. For GsMTx‐4 (Alomone Labs, Jerusalem, Israel) replicates to inhibit Piezo1, cells were pretreated with 10 µm of the inhibitor, and incubated for 1 h on a rotator prior to ultrasound exposure. Some samples were pretreated with 2 mm EGTA (Bioworld) for 30 min on a rotator before ultrasound exposure. For 2‐APB (Tocris Bioscience, Bristol, BS11 9QD, UK) treatment to inhibit TRP channels, PC3 cells were pretreated with 10, 50, or 100 µm of the inhibitor and incubated for 30 min on a rotator prior to the ultrasound exposure.

### In Vitro Ultrasound Parameters and Phantom Simulation

The ultrasound parameters for the in vitro experiments consisted of a 1.1 MHz transducer, 60 or 120 s exposure time, pressure ranging from 263–1993 kPa, 0.33 Hz PRF, 17% DC and burst duration of 500 ms. These parameters were chosen based on their success in activating mechanosensitive ion channels in prior studies of neurons.^[^ ^128^ ^]^ The transducer was an H101 single element focused transducer (Sonic Concepts) with a radius of curvature of 63.2 mm and a center frequency of 1.1 MHz. It was powered by an arbitrary waveform generator and amplifier (33500B, Keysight Technologies, Santa Rosa, CA, USA; A150, Electronics & Innovation, Rochester, NY, USA). The transducer was connected to a 3D‐printed waveguide capable of holding samples at the focus. The transducer was calibrated using a fiber optic hydrophone (Precision Acoustics) that was moved through the pressure field generated inside a water‐filled cuvette. The fiber optic hydrophone was also used to measure the temperature change at the ultrasound focus over the course of a 1‐ and 2‐min treatment (Figure S3, Supporting Information). The mechanical index was estimated as the peak rarefactional pressure divided by the square root of the center frequency, as shown in Equation (1).^[^ ^42^ ^]^

(1) $$\mathrm{MI}\;=\frac{\mathrm{Peak}\;\mathrm{Rarefactional}\;\mathrm{Pressure}}{\sqrt{\mathrm{Frequency}}}\;$$

where the peak rarefactional pressure is in MPa and the frequency is in MHz. For instance, at a pressure of 944 kPa (0.944 MPa) and frequency of 1.1 MHz, the MI is calculated to be 0.9.

It is noted that in some later experiments, a very similar ultrasound apparatus established at Rice University was used. The same arbitrary waveform generator (33500B, Keysight Technologies), amplifier (A150, Electronics & Innovation), and 1.1 MHz transducer (H‐101, Sonic Concepts) were used as in prior experiments conducted at Vanderbilt University. The driving signal from the function generator was amplified by a radio frequency power amplifier with a 55 dB gain. The amplified signal was then routed through a matching network and delivered to a 1.1 MHz FUS transducer. The cell suspension in a microcentrifuge tube was exposed to pulsed FUS stimulation (0.33 Hz PRF, 17% DC, and 500 ms burst duration) for either 60 or 120 s as described above, matching the parameters described in Figure 1B. To match the low (263 kPa) or high (944 kPa) FUS pressures used in previous studies (Figures 2, 3, 4, 5), a fiber‐optic hydrophone (HFO‐690, ONDA, USA) was employed to measure the peak negative pressure. A motorized 2D translation stage was then used to scan the acoustic pressure in the sample plane perpendicular to the FUS propagation direction to target the cell suspension as depicted in Figure 1A.

### Phantom Simulation of In Vivo Ultrasound Parameters

A cylindrical 40 g phantom composed of 1% agarose, 4% graphite, and 95% water was created with a fiber optic hydrophone suspended in the midsection of the phantom to simulate the acoustic conditions of the tissue transducer was attached to a waveguide with an opening at the geometric focus which was the filled with 1% agarose gel. The waveguide was then coupled to the phantom using FUS gel. The phantom was adjusted to find the focal position of the transducer and allowed to reach a steady temperature. The phantom was sonicated with 500 ms pulses at a frequency of 1.1 MHz and a pressure of 944 kPa. The pulses were repeated at a frequency of 0, 0.1, and 0.2 Hz for 10 min, with 20 min between sonications. These were repeated twice to ensure accuracy. The temperature change was recorded by the fiber optic hydrophone. The temperature measurements were verified with a thermal acoustic simulation performed in the MATLAB package KWave (Figure S9, Supporting Information).^[^ ^129^ ^]^

### Generation of the Piezo1 shRNA Knockdown Cell Line

To knockdown *PIEZO1*, shRNA lentiviral particle transduction from Santa Cruz Biotechnology was used. On day 1, 5 × 10^4^ PC3 cells were plated in a 12‐well plate and incubated at 37 °C for 24 h prior to viral infection, so that cells were ≈50% confluent on the day of infection. On day 2, 5 µg mL^−1^ Polybrene (Santa Cruz Biotechnology, Dallas, TX, USA) was added with fresh complete media to each well that was to be transduced. Cells were infected by adding 15 µL of shRNA lentiviral particles against either *PIEZO1* (shPiezo1) [sc‐93227‐v] or the control (shC) [sc‐108080]. The plate was placed on a rocker for 10 min and then incubated at 37 °C overnight. On day 3, the media was removed, and 1 mL of fresh complete media (without polybrene) was added, and the cells were incubated overnight at 37 °C. On day 4, stable clones expressing the shPiezo1 were lifted using 0.25% trypsin‐EDTA and moved to a 6‐well plate. Stable clones expressing shPiezo1 and shC were selected using puromycin dihydrochloride (Fisher Scientific). For selection, media was changed every 3–4 days, while increasing the concentration of puromycin from 5 to 15 µg mL^−1^.

### PIEZO1 shRNA Knockdown Validation

Knockdown of Piezo1 in the PC3 cells was validated using flow cytometry. PC3 cells were washed in HBSS with Ca^2+^ and Mg^2+^ (HBSS++) (Corning) and lifted using 0.25% trypsin‐EDTA for 5–6 min at 37 °C. Cells were spun at 300×*g* to remove dissociation agent and resuspended in HBSS++ at a concentration of 0.5 × 10^6^ cells mL^−1^ prior. 1 × 10^6^ cells were pipetted into 1.5 mL microcentrifuge tubes (Corning) for flow cytometry preparation. Cells were fixed with 4% paraformaldehyde (PFA) (Electron Microscopy Sciences, Hatfield, PA, USA) aqueous solution in HBSS++ (v/v) and incubated at room temperature (RT) for 15 min. The cells were washed twice in HBSS++, centrifuged at 800×*g* for 5 min, followed by permeabilization with 100% ice cold methanol, and incubated on ice for 10 min. The cells were washed twice in HBSS++, centrifuged at 800×*g* for 5 min. The samples were incubated for 30 min in the dark in 100 µL of 1% bovine serum albumin (BSA) (Sigma‐Aldrich) in HBSS++, with either Alexa Fluor 488 anti‐Piezo1 antibody (NBP1‐78446AF488) (Novus Biologicals, Centennial, CO, USA) or Alexa Fluor 488 Rabbit IgG isotype control [NBP2‐24982] (Novus Biologicals).

The cells were washed once in 1% BSA and centrifuged at 800×*g* for 5 min. To prepare for flow cytometry analysis, the cells were resuspended in 250 µL of HBSS++. The green‐blue (525 nm emission, 488 nm excitation) laser was used to determine the percentage of PC3 cells expressing Piezo1 for the parental, shC, and shPiezo1 samples.

### Annexin‐V Cellular Apoptosis Assay

To assess cell apoptosis and necrosis, FITC‐conjugated Annexin‐V (AV) (BD Pharmingen, San Diego, CA, USA) and propidium iodide (PI) (BD Pharmingen) were used. The manufacturer's instructions were followed to prepare samples for analysis via flow cytometry. After 24 h, media from each well was transferred to microcentrifuge tubes. Cells were detached from the 24‐well plate by incubating with 0.25% trypsin‐EDTA at 37 °C for 5–6 min. Complete media was added to neutralize the solution and collect the samples from each well to transfer to respective microcentrifuge tubes. Cells were centrifuged at 300×*g* for 5 min and washed one time in HBSS++. Cells were incubated with AV and PI antibodies at RT for 15 min in the absence of light, followed by immediate flow cytometry analysis.

Viable cells were identified as being negative for Annexin‐V and PI, necrotic cells were positive for PI, early apoptotic cells were positive for Annexin‐V, and late apoptotic cells are positive for Annexin‐V and PI. Control samples were used with each experiment to calibrate the instrument: cell samples labeled with Annexin‐V or PI to define boundaries of the cell populations, and unlabeled samples in HBSS++ to quantify the level of autofluorescence.

TRAIL sensitization was calculated using the following equations:

(2) $$\mathrm{TRAIL}\;\mathrm{Sensitization}\;=\frac{\left(\%\mathrm{Cells}-\%\mathrm{Cells},\mathrm{TRAIL}\right)}{\%\mathrm{Cells}}$$

(3) $$\mathrm{TRAIL}\;\mathrm{Sensitization}\;\left(\mathrm{FUS}\right)=\frac{\left(\%\mathrm{Cells},\mathrm{FUS}-\%\mathrm{Cells},\mathrm{FUS},\mathrm{TRAIL}\right)}{\%\mathrm{Cells},\mathrm{FUS}}$$

(4) $$\begin{array}{ccc} & & \mathrm{TRAIL}\;\mathrm{Sensitization}\;\left(\mathrm{GsMTx}4\right)\\ & & \;=\frac{\left(\%\mathrm{Cells},\mathrm{FUS},\mathrm{GsMTx}4-\%\mathrm{Cells},\mathrm{FUS},\mathrm{GsMTx}4,\mathrm{TRAIL}\right)}{\%\mathrm{Cells},\mathrm{FUS},\mathrm{GsMTx}4} \end{array}$$

(5) $$\begin{array}{ccc} & & \mathrm{TRAIL}\;\mathrm{Sensitization}\;\left(\mathrm{EGTA}\right)\\ & & \;=\frac{\left(\%\mathrm{Cells},\mathrm{FUS},\mathrm{EGTA}-\%\mathrm{Cells},\mathrm{FUS},\mathrm{EGTA},\mathrm{TRAIL}\right)}{\%\mathrm{Cells},\mathrm{FUS},\mathrm{EGTA}} \end{array}$$

This was based on the percentage of viable populations determined using the AV/PI assay.

*JC‐1 assay*: After 24 h, media from each well was transferred to microcentrifuge tubes. Cells were detached from the 24‐well plate by incubating with 0.25% trypsin‐EDTA at 37 °C for 5–6 min. Complete media was added to neutralize the solution and collect the samples from each well to transfer to respective microcentrifuge tubes. The manufacturer's directions were followed for the JC‐1 (Abcam, Cambridge, MA, USA) assay. Briefly, the cells were centrifuged at 300×*g* for 5 min and washed thoroughly with HBSS. Samples were then incubated with 10 µm JC‐1 dye for 30 min in the dark at 37 °C. Cells were centrifuged at 300×*g* for 5 min, washed in HBSS, and JC‐1 fluorescence was analyzed via flow cytometry using the red‐violet and green–violet lasers. Cells with depolarized mitochondria were identified as populations with decreased JC‐1 red fluorescence, and cells with healthy mitochondria were identified by having high red fluorescence.

Some of the same AV/PI and JC1 replicates for the PC3 cells at 263 and 944 kPa, 60 s FUS exposure are used in Figures 2 and 3. Some of the same replicates for the PC3 cells at 380 kPa, 60 s FUS exposure are used in Figure S1 (Supporting Information) and Figure 3.

### Human Apoptosis Array Proteome Profiler

PC3 cells were lifted with 0.25% trypsin‐EDTA and exposed to TRAIL and FUS treatments as described above. The samples were placed in 100 mm petri dishes (229695, Cell Treat, Ayer, MA, USA) and incubated at 37 °C for either 4 or 24 h to be used for the Proteome Profiler Human Apoptosis Array Kit (ARY009, R&D Systems, Minneapolis, MN, USA) to analyze protein expression. To collect supernatant, dead cells were first collected and washed once in HBSS++. Meanwhile, the remaining adherent cells were lysed directly from the petri dish using a cell scraper and added to the dead cell pellet previously collected to provide a lysate containing both live and dead cells. Briefly, lysates were placed on a rocker at 4 °C for 30 min, then centrifuged at 14 000×*g* for 5 min. The supernatant samples were transferred to clean microcentrifuge tubes for analysis via the Proteome Profiler Array Kit following the manufacturer's instructions. The ChemiDoc Imaging System (12003153, BioRad, Hercules, CA, USA) was used to capture the chemiluminescence exposure and FIJI image software was used to quantify protein expression. Mean pixel intensity has been corrected via background subtraction per each membrane.

### DR4/5 Expression via Confocal Imaging

For microscopy, PC3 cells were lifted from culture using 0.25% trypsin‐EDTA as described in the Cell Preparation section above. Cells were fixed in 4% PFA aqueous solution in HBSS with Ca^2+^ and Mg^2+^ (v/v) and incubated for 15 min. The cells were washed twice in HBSS++, followed by permeabilization using Triton X‐100 (Sigma‐Aldrich) diluted to 1× in HBSS++ (v/v) at RT for 10 min. Samples were blocked in 5% BSA and 5% goat serum (diluted from 10% Normal Goat Serum (Life Technologies, Carlsbad, CA)) (v/v) for 45 min at RT. Cells were incubated in either DR4 polyclonal antibody [PA5‐92857] (Fisher Scientific) or TRAIL‐R2 (DR5) recombinant rabbit monoclonal antibody (JA03‐38) [MA5‐32693] (Fisher Scientific), and diluted at 1:100 (v/v) in 1% BSA for 1 h. Samples were stained with goat anti‐rabbit IgG (H+L) Alexa Fluor 488 secondary antibody [A11008] (Invitrogen), 1 µg mL^−1^ DAPI nuclear stain (BD Pharmingen), and ActinRed 555 ReadyProbes Reagent (Invitrogen) for 30 min in the absence of light. Washing steps were performed between each step and all incubation steps were completed at RT. The stained cells were spun onto glass slides using a CytoSpin3 (Shandon). A drop of VECTASHIELD antifade mounting media (H‐1000, Vector Laboratories) and coverslips were added. Slides were imaged on an LSM 900 Zeiss confocal microscope equipped with a 40× oil immersion objective.

FIJI image software was used to measure the percentage of DR4/5 expression within each cell, using an in‐house developed macro. The corrected total cell fluorescence (CTCF) was calculated using the following equation:

(6) $$\begin{array}{c} \mathrm{CTCF}=\mathrm{Integrated}\;\mathrm{Density}-\left(A_{\mathrm{selected}\;\mathrm{cell}}\times \mathrm{Mean}\;\mathrm{fluorescenc}e_{\mathrm{background}}\right) \end{array}$$

where “*A*” is the area of the selected cell.

### DR4/5 Antibody Verification

To determine if the DR4/5 antibodies used for the IHC staining of samples collected from the in vivo studies were in fact labeling the extra‐ and/or intra‐cellular regions of the DR4/5 receptors, flow cytometry was used to compare staining methods. PC3 cells were washed in HBSS++ and lifted using 0.25% trypsin‐EDTA for 5–6 min at 37 °C. Cells were spun at 300×*g* to remove dissociation agent and resuspended in HBSS++ at a concentration of 0.5 × 10^6^ cells mL^−1^ prior. 1 × 10^6^ cells were pipetted into 1.5 mL microcentrifuge tubes for flow cytometry preparation. All samples were fixed with 4% PFA aqueous solution in HBSS++ (v/v) and incubated at RT for 15 min. The cells were washed twice in HBSS++ and centrifuged at 800×*g* for 5 min.

Next, half the samples were permeabilized with 100% ice cold methanol, incubated on ice for 10 min, and then washed twice in HBSS++, and centrifuged at 800×*g* for 5 min. The other half of the samples were fixed without permeabilization. All samples were incubated for 30 min in 100 µL of 1% BSA, with either DR4 polyclonal antibody [PA5‐92857], TRAIL‐R2 (DR5) recombinant rabbit monoclonal antibody (JA03‐38) [MA5‐32693] or remained unstained. The cells were washed once in 1% BSA and centrifuged at 800×*g* for 5 min. Samples were then stained with goat anti‐rabbit IgG (H+L) Alexa Fluor 488 secondary antibody [A11008] or Alexa Fluor 488 rabbit IgG isotype control [NBP2‐24982] for 30 min in the dark in 100 µL of 1% BSA.

The cells were washed once in 1% BSA and centrifuged at 800×*g* for 5 min. To plate for flow cytometry analysis, the cells were resuspended in 250 µL of HBSS with Ca^2+^ and Mg^2+^. The green‐blue (525 nm emission, 488 nm excitation) laser was used to determine the percentage of PC3 cells expressing DR4/5 for the fixed, or fixed and permeabilized conditions.

### Subcutaneous Tumor In Vivo Studies

Male eight‐week‐old nude (24102262‐NU/NU) mice were obtained from Charles River Laboratory (Wilmington, MA, USA). This study was approved by the Institutional Review Board protocol #M1700009‐02. All mice received identical care and were maintained in sterile housing conditions. Mice were monitored by veterinary staff in the Vanderbilt University Division of Animal Care (DAC).

PC3 cells were lifted and counted with a hemocytometer. Cells were washed twice with HBSS at 300×*g* for 5 min. 1 × 10^6^ PC3 cells were resuspended in a 250 µL mixture of 1:1 (v/v) phosphate buffered saline (PBS) (Corning) and Matrigel (Corning Life Sciences, Tewksbury, MA, USA). Both flanks on the mice were inoculated with 1 × 10^6^ cells using 29 G needles (Exel International, CA, USA) while the mice were anesthetized. Tumors were measured using calipers 2–3 times per week while the mice were anesthetized.

During the in vivo studies, the ultrasound transducer was coupled to a 3D‐printed waveguide filled with 1.5% agarose. The focal distance of the transducer was 63.2 mm. The length of the focus, calculated as the volume where the pressure was greater than ½ the maximum pressure, was 10.21 mm and the width was 1.37 mm. The waveguide was designed to open at the acoustic focus. The opening of the waveguide was aligned with the tumor on the mouse and coupled with ultrasound gel. Before sonication the alignment of the tumor and the transducer focus were visually confirmed and the gel was checked to ensure that no bubbles were present. The mice received a single ultrasound exposure per treatment, 5 min for the single treatment study and 10 min for the multi‐dose study, with a constant ultrasound pressure of 944 kPa. The treatment time was adjusted to ensure that the TRAIL had sufficient time to perfuse through the tumor adequately and the PRF lowered to avoid thermal effects. Thermal simulations and phantom experiments indicated that a PRF of 0.2 Hz could generate biologically significant heating in vivo (Figure S9, Supporting Information). The duty cycle, frequency and burst duration remained the same as the in vitro parameters. A single TRAIL dosage consisted of 2 mg kg^−1^ mouse weight, injected locally into the tumors using a 29 G needle. Mice remained under isoflurane during the ultrasound exposure, while their tumor was positioned over the focal point of the transducer.

For the single treatment study, the tumors were inoculated on day –7 and the treatment was administered on day 0. Tumors were resected on day 21 for IHC analysis. For the multi‐dose in vivo study in Figure 6, tumors were inoculated on day –14 and the first treatment given on day 0, followed by the second treatment on day 6. Mice were euthanized on day 48 post‐tumor cell inoculation and immediately processed for IHC. The PBS vehicle control was delivered intratumorally (100 µL) using a 29 G needle. For the varied treatment intervals (Figure 7) between the TRAIL injection and ultrasound exposure, the tumors were inoculated on day –13, and treatments were performed on day 0 and day 8. Mice were euthanized and tumors were resected on day 49. Mice were euthanized at humane endpoints, as determined by the tumor size and recommendation by DAC veterinary staff.

Tumor volume was measured using the following, where “*L*” is the length and “*W*” is the tumor width:

(7) $$\mathrm{Tumor}\;\mathrm{volume}\;=\frac{L\cdot W^{2}}{2}$$

Tumor growth rate was measured using the equation below:

(8) $$\mathrm{Tumor}\;\mathrm{growth}\;\mathrm{rate}=\frac{\left(\mathrm{Tumor}\;\mathrm{vo}l_{t_{f}}-\mathrm{Tumor}\;\mathrm{vo}l_{t_{i}}\right)}{\mathrm{Days}\;\mathrm{elapsed}}$$

where *t*_f_ is the final tumor volume and *t*_i_ is the initial tumor volume.

### Immunohistochemistry Staining and Analysis

Tumors were immediately fixed in 10% neutral buffered formalin (NBF) for 48 h following tumor resection. The Vanderbilt Translational Pathology Shared Resource routinely processed, paraffin embedded, and sectioned the tumors at 5 µm. The tumor sections were placed on slides, and stained for cleaved caspase‐3, DR4, DR5, E‐cadherin, Ki‐67, pan‐cytokeratin, Piezo1, and vimentin using the Leica Bond‐RX staining platform. Briefly, all steps besides dehydration, clearing and coverslipping were performed on the Leica Bond stainer. Slides were deparaffinized. Heat induced antigen retrieval was performed on the Bond stainers as follows: Epitope Retrieval 2 solution for 10 min (Piezo1, vimentin), 15 min (E‐cadherin), or 20 min (Ki67, DR4), or using Dako's Proteinase K (S3020, DAKO/Agilent, Carpinteria, CA) for 5 min (pancytokeratin). Slides were incubated with the primary antibodies as follows and only single staining was performed: anti‐E‐Cadherin [AF748] (R&D Systems) for 1 h at a 1:1000, Caspase‐3 [9664] (Cell Signaling) diluted 1:300 for 1 h, anti‐Vimentin [2707‐1] (Epitomics, Burlingame, CA) diluted 1:3000 for 1 h, pan‐Cytokeratin [Z0622] (Dako, Carpinteria, CA) diluted 1:4000 for 1 h, anti‐Ki67 [12202S] (Cell Signaling Technology, Danvers, MA) diluted 1:1000 for 1 h, anti‐Piezo1 [15939‐1AP] (ProteinTech, Rosemont, IL) at a 1:500 dilution for 1 h, anti‐DR4 [PA5‐92857] (Invitrogen/ThermoFisher, Kalamazoo, MI) at a 1:1000 dilution for 1 h, and anti‐DR5 [MA5‐32693] (Invitrogen/ThermoFisher, Kalamazoo, MI) at a 1:2000 dilution for 1 h. E‐cadherin primary antibody was followed by a biotinylated anti‐goat antibody [BA‐5000] (Vector Laboratories, Inc.) for 30 min at a 1:2000 dilution. The Bond Polymer Refine detection system was used for visualization. For TUNEL staining slides were placed on the Leica Bond RX IHC stainer. All steps besides dehydration, clearing and coverslipping are performed on the Bond RX. Slides are deparaffinized. Antigen retrieval was performed on the Bond RX using Triton X‐100 (9284, St. Louis, MO) for 5 min. Slides were incubated with Equilibration Buffer (G7130, Promega, Madison, WI) for 5 min, followed with the TdT reaction mix (G7130, Promega, Madison, WI) for 10 min, and SSC‐x20 (G7130, Promega, Madison, WI) for 10 min. The Bond Intense R detection system (DS9263, Leica, Buffalo Grove, IL) was used for visualization. All slides were then dehydrated, cleared and coverslipped. Slides were then scanned and uploaded to SlideViewer (3DHistotech) to acquire digital images for analysis.

FIJI was used to quantify DAB positive staining expression and the percentage of proliferating cells using in‐house developed macros. The color deconvolution function was applied to the snapshots to provide an H&E, DAB, and residual image, so that area fractions (percent positive) could be determined using the DAB image. Data are presented as the percent positive DAB stain ± standard error of the mean of two sections per tumor per IHC stain. A board‐certified veterinary pathologist examined H&E‐stained tumor slides and did not find any obvious morphologic differences between the untreated and treated tumors.

### Flow Cytometry

All flow cytometry experiments were completed using a Guava easyCyte 12HT benchtop flow cytometer (MilliporeSigma). Flow cytometry gating and analysis was completed using FlowJo software (FlowJo, Ashland, OR, USA).

### Statistics and Reproducibility

Data are reported as mean and standard error of the mean. Experiments included at least 2–3 technical replicates per each biological replicate, with at least *n* *=* 3 biological replicates completed. Statistical significance is indicated as: *p < 0.05, **p < 0.01, ***p < 0.005, and ****p < 0.001 for significance; otherwise, no significant difference was found. GraphPad Prism software was used to perform statistical analyses and produce figures for this article.

### Ethics Approval

The protocol for all mouse studies in this article was approved by the Vanderbilt IACUC protocol #M1700009‐02 and mice were monitored by staff from the Division of Animal Care (DAC) at Vanderbilt University.

## Conflict of Interest

The authors declare no conflict of interest.

## Author Contributions

A.R.F. and M.R.K. conceived of this study. A.R.F. wrote the initial draft of this article and completed the formal analysis. A.R.F., M.W.N., C.F.C., and M.R.K. developed conceptualization and methodology. A.R.F., M.W.N., S.J.R., S.V.K., J.A.D., B.G.K., and K.N.G.C. conducted the investigation. A.R.F., M.W.N., S.J.R., J.A.D., and J.K. completed mouse procedures. M.W.N. and L.R. supplied the ultrasound apparatus and performed sonications for in vitro and in vivo studies. M.R.K. and C.F.C. supervised and edited the work.

## Supporting information



Supporting Information

## Data Availability

All data needed to evaluate the conclusions in the paper are present in the paper and/or the Supplementary Materials.
