# Supplementary material for: Applying Ultrasound to Mechanically and Noninvasively Sensitize Prostate Tumors to TRAIL‐Mediated Apoptosis
Source: Adv Sci (Weinh). 2025 Feb 20;12(15):2412995. doi: 10.1002/advs.202412995 (PMC12005757; doi:10.1002/advs.202412995)
Supplement: Supplementary file 1 — Supporting Information [file ADVS-12-2412995-s001.docx]

Supporting Information

Applying ultrasound to mechanically and noninvasively sensitize prostate tumors to TRAIL-mediated apoptosis

*Abigail R. Fabiano^1,2^, Malachy W. Newman^1^, Jenna A. Dombroski^1^, Schyler J. Rowland^1^, Samantha V. Knoblauch^1^, Jiro Kusunose^3^, Katherine N. Gibson-Corley^4^, Benjamin G. Kaufman^2^, Liqin Ren^2^, Charles F. Caskey^1,5^, Michael R. King^1,2*^.*


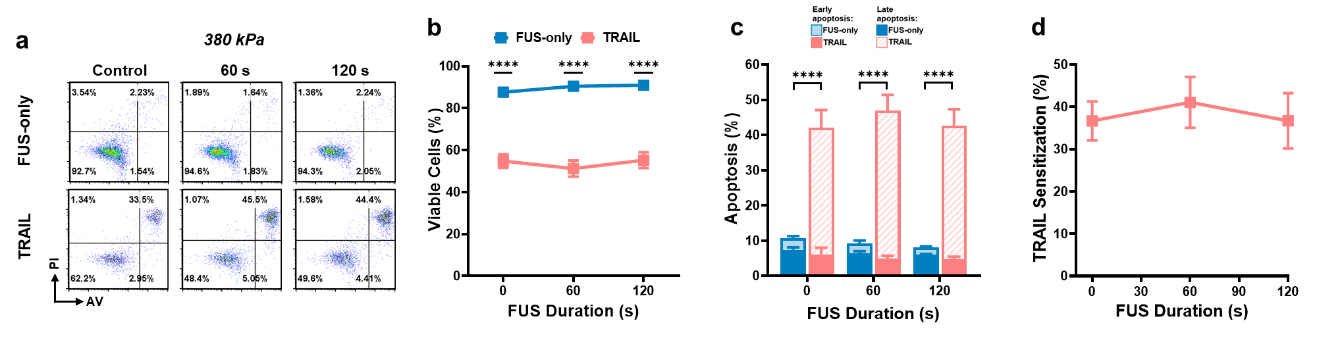


**Figure S1. Doubling the FUS duration had no effect on apoptosis at low pressure.** (a) Representative AV/PI flow cytometry plots. Mean percentage of (b) viable cells, (c) apoptotic cells and (d) TRAIL sensitization (Equation 2 and 3) for PC3 cells exposed to a 380 kPa ultrasound stimulus for 60 or 120 s. *n* = 3 independent experiments; two-way ANOVA (b,c), and unpaired t-test comparing the TRAIL sensitization at each pressure to the no-ultrasound condition (d). *p<0.05, ****p<0.0001. Error bars represent mean ± SEM.

**Figure S2. Doubling the FUS duration has no significant effect on mitochondrial depolarization.** PC3 cells were treated with 200 ng/mL TRAIL and exposed to a 380 kPa ultrasound for 0, 60 or 120 s. Summary data for the mean percentage of depolarized mitochondria. *n* = 3 independent experiments; two-way ANOVA. *p<0.05. Error bars represent mean ± SEM.


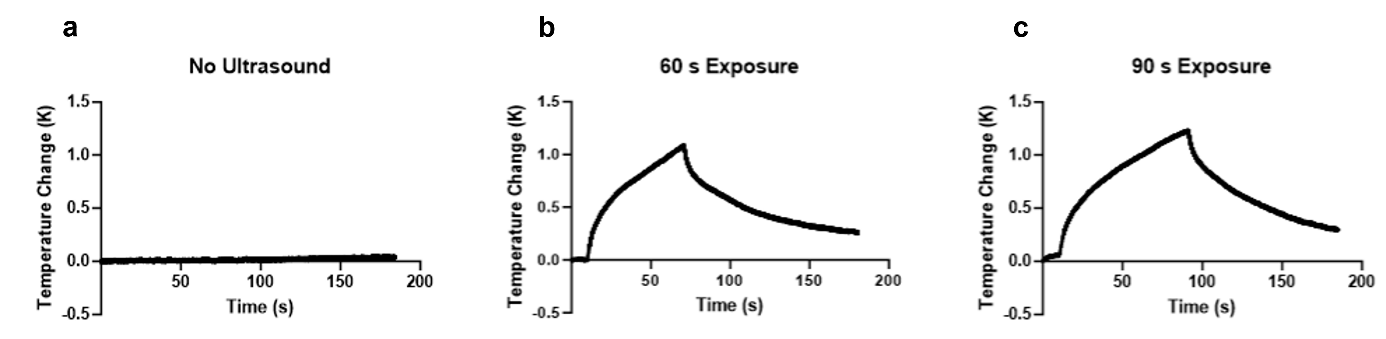


**Figure S3. In vitro phantom simulation of ultrasound pressure.** Changes in temperature in vitro for (a) no ultrasound exposure and for a (b) 60 s and (c) 90 s ultrasound exposure. The FUS consisted of 500 ms pulses at a pressure of 944 kPa repeating at a frequency of 0.33 Hz.


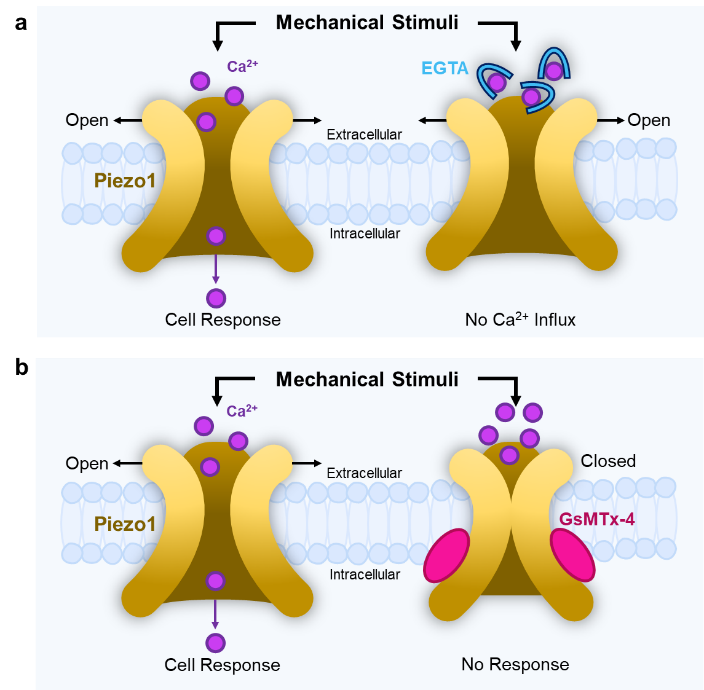


**Figure S4. Schematic representing the mechanical effects of Ca^2+^ chelation and Piezo1 inhibition.** (a) EGTA chelates free Ca^2+^ that cells are exposed to, inhibiting Ca^2+^ influx into cells. (b) GsMTx-4 inhibits Piezo1 activation, holding the beams closed when stimulated.


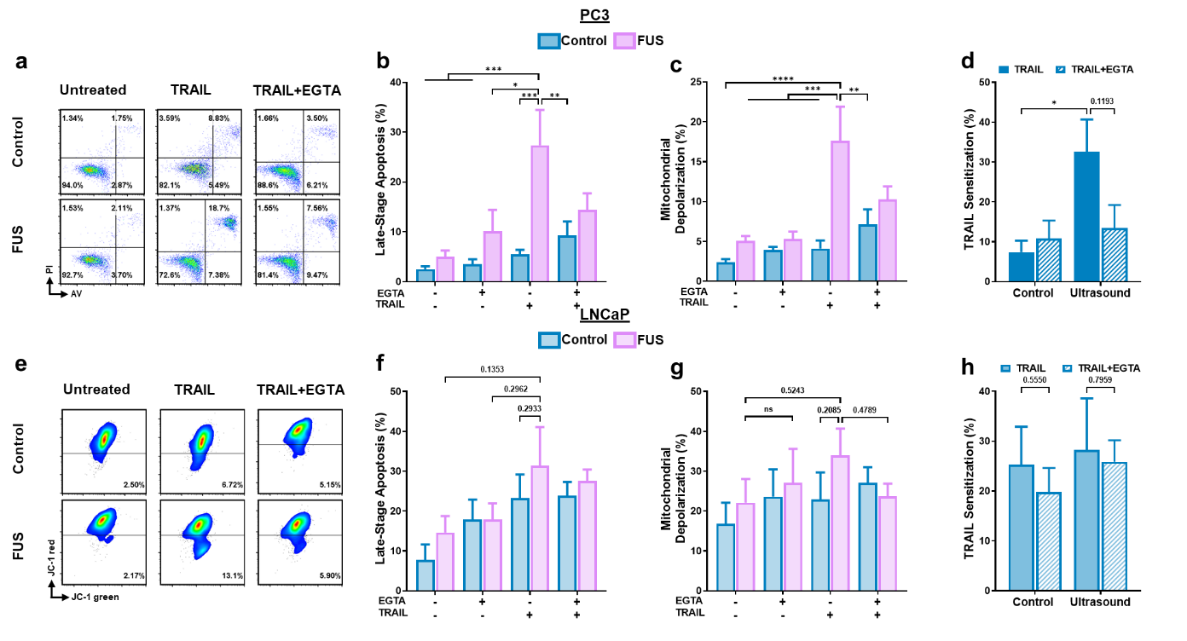


**Figure S5. Ca^2+^ chelation reduces intrinsic apoptosis.** (a) Representative AV/PI flow cytometry plots showing PC3 cells. Summary data for the mean percentage of (b) late-stage apoptosis, (c) mitochondrial depolarization and (d) TRAIL sensitization for PC3 cells (Equation 2, 3 and 5). (e) Representative JC-1 flow cytometry plots showing PC3 cells. Summary data for the mean percentage of (f) late-stage apoptosis, (g) mitochondrial depolarization and (h) TRAIL sensitization (Equation 2, 3 and 5) observed in LNCaP cells. PCa cells were exposed to 60 s of ultrasound at 944 kPa, treated with TRAIL, EGTA and ultrasound. *n* = 3-5 independent experiments; two-way ANOVA (b-d, f-h). *p<0.05, **p<0.01, ***p<0.005, ****p<0.0001. Error bars represent mean ± SEM.

**
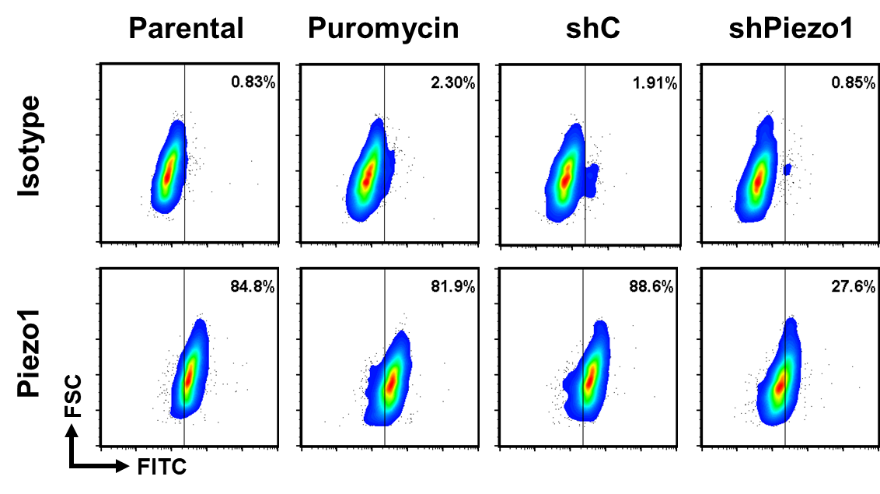
**

**Figure S6. Validation of Piezo1 knockdown in PC3 cells.** Flow cytometry validation shows a decrease in Piezo1 expression for the shPiezo1 PC3 cells compared to untreated (parental) cells, puromycin and shC PC3 cells (FSC = forward scatter).

**
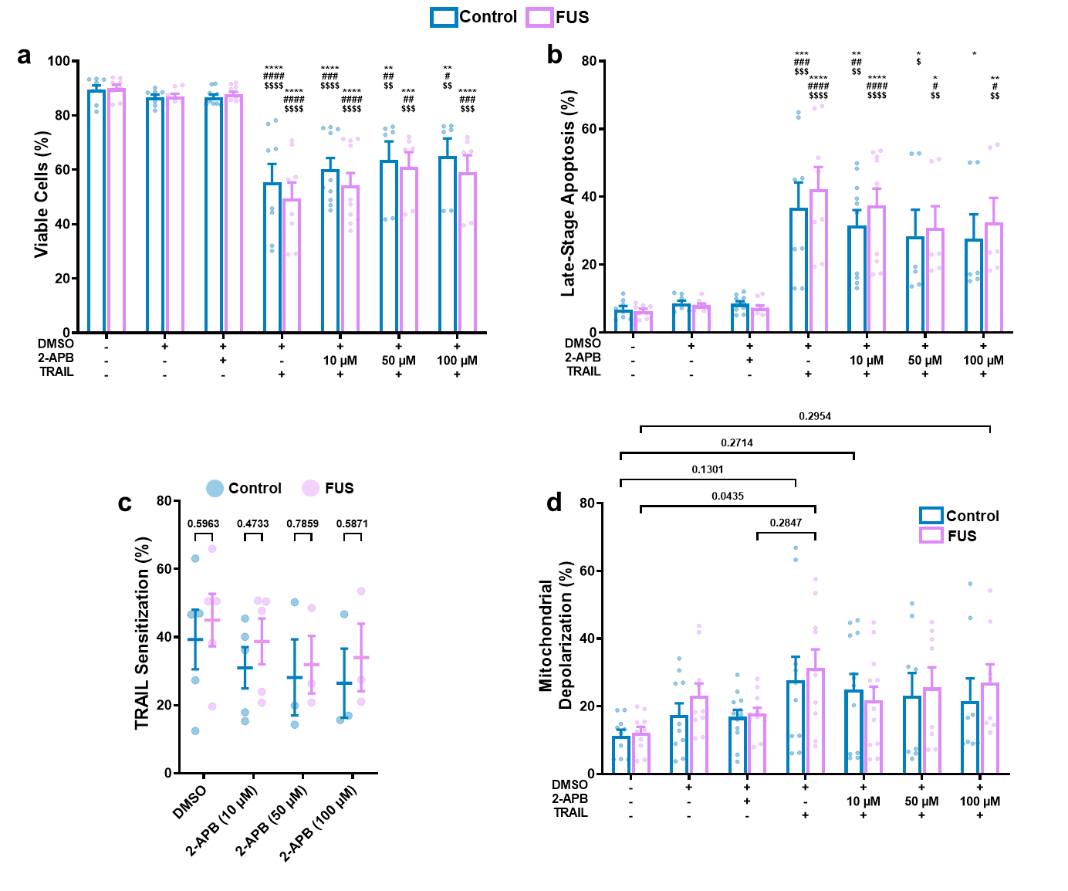
**

**Figure S7. TRP channel inhibition in PC3 cells.** Summary data for the mean percentage of (a) viable, (b) late-stage apoptosis, (c) TRAIL sensitization and (d) mitochondrial depolarization for PC3 cells treated with TRAIL, 2-APB and ultrasound at 944 kpa for 60 s. *n* = 3 independent experiments; two-way ANOVA. (*) shows the statistical comparison to the untreated group, (#) shows the statistical comparison to the DMSO-treated group and ($) shows the statistical comparison to the DMSO+2-APB-treated group for the respective control or FUS conditions. The statistical values for all symbols correspond to; *p<0.05, **p<0.01, ***p<0.005, ****p<0.0001. Error bars represent mean ± SEM.


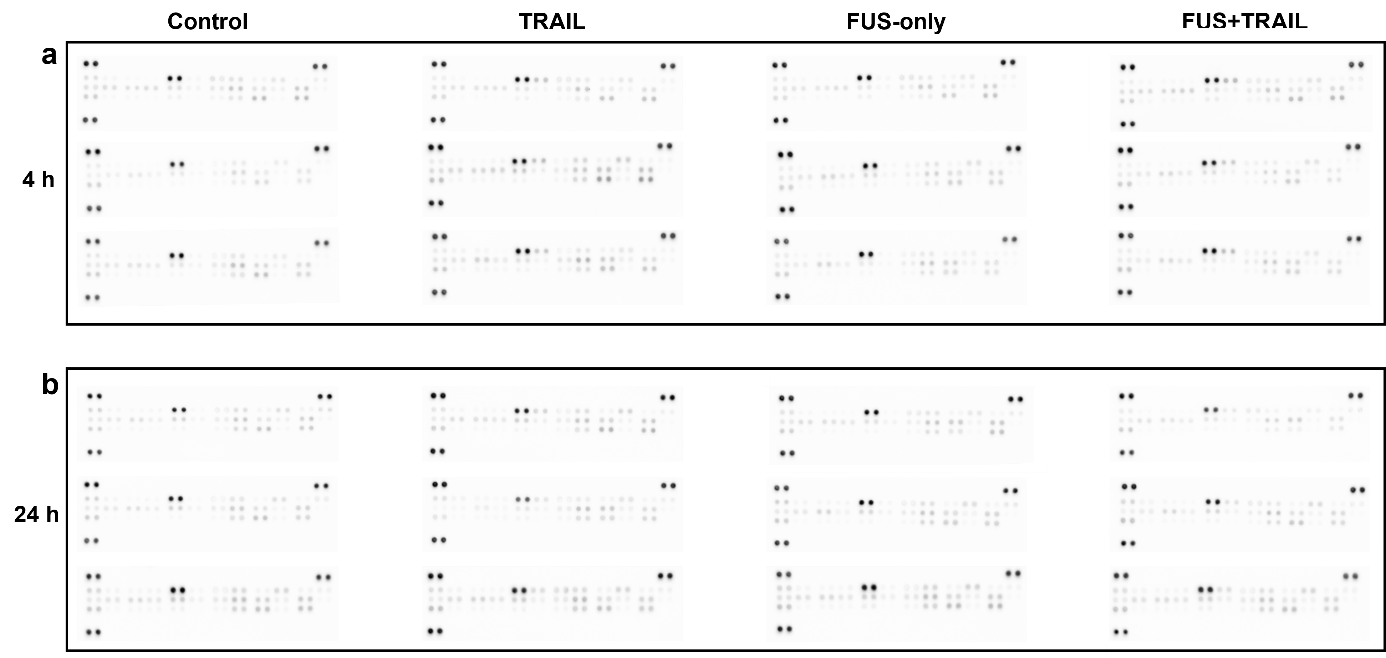


**Figure S8. Proteome profiler membranes.** Apoptosis array protein expression of PC3 cells captured via chemiluminescence imaging at (a) 4 and (b) 24 h post-treatment.


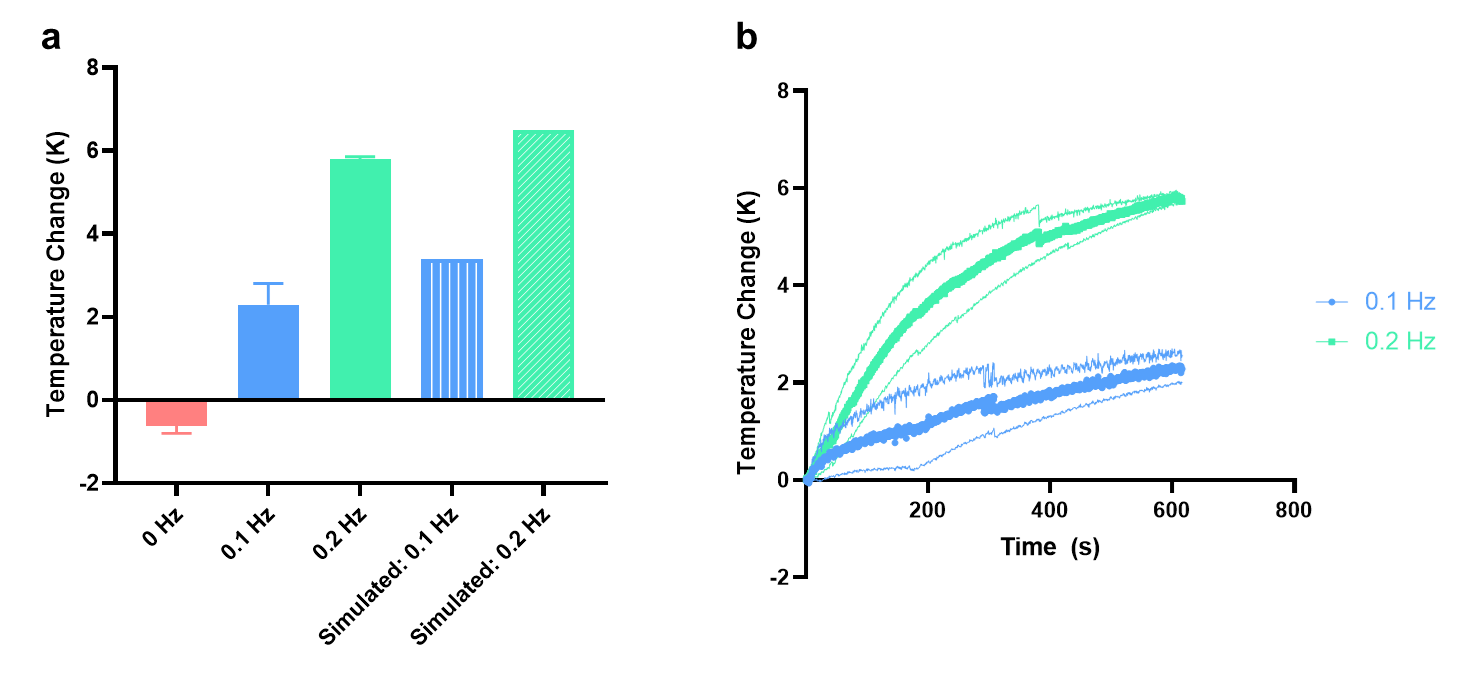


**Figure S9. In vivo ultrasound phantom simulation.** (a) Average temperature changes for 10 min and (b) temperature change as a function of time for ultrasound pulses at a rate of 0, 0.1 and 0.2 Hz. Simulated refers to numerical experiments completed in MATLAB. *n* = 3 independent experiments. Error bars represent mean ± SEM.


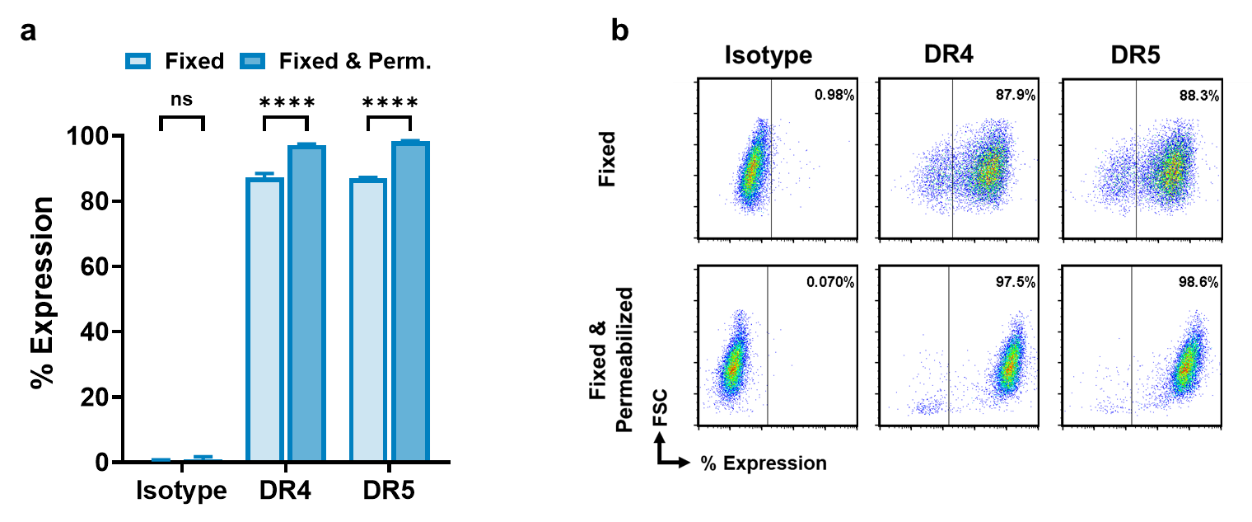


**Figure S10. DR4/5 antibody binding.** (a) Summary data for the mean percentage of DR4/5 expression in PC3 cells that are fixed (4% PFA) or fixed and permeabilized (4% PFA + 100% ice-cold methanol). (b) Representative flow cytometry plots for the data in a. *n* = 3 independent experiments; two-way ANOVA (a). ****p<0.0001. Error bars represent mean ± SEM.


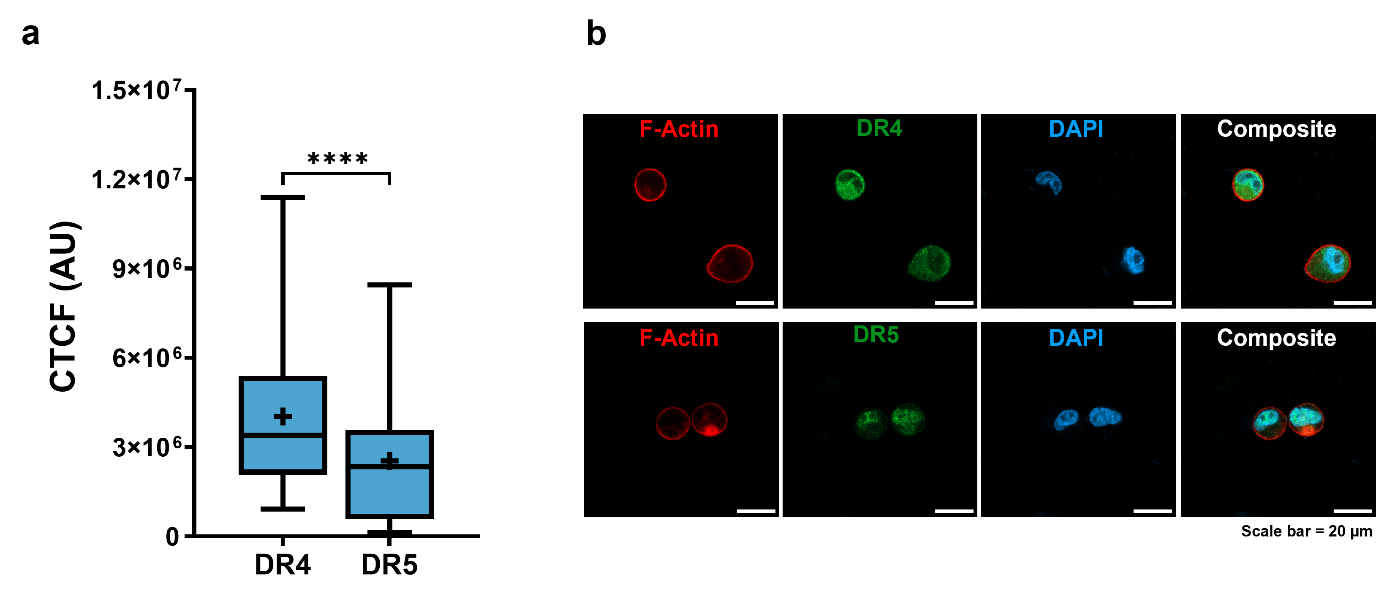


**Figure S11. Confocal analysis of DR4/5 expression.** (a) Corrected total cell fluorescence (CTCF) of DR4/5 expression in PC3 cells acquired via confocal imaging (Equation 6). (b) Representative micrographs of F-actin, DR4/5 and DAPI expression. Box and whisker plots extend from min to max, mean indicated by (+) and median indicated by solid, horizontal line within the box. *n* = 3 independent experiments; *n* = 159 cells for DR4, *n* = 183 cells for DR5; unpaired *t*-test (a). ****p<0.0001.

**Figure S12. Piezo1 expression in normal and tumor prostate tissue.** Comparison of Piezo1 gene expression using non-paired data adapted from tnmplot.com.^[1]^ Unpaired *t*-test, ***p<0.005. Box and whisker plot extends from min to max, mean indicated by (+) and median indicated by solid, horizontal line within the box.

**
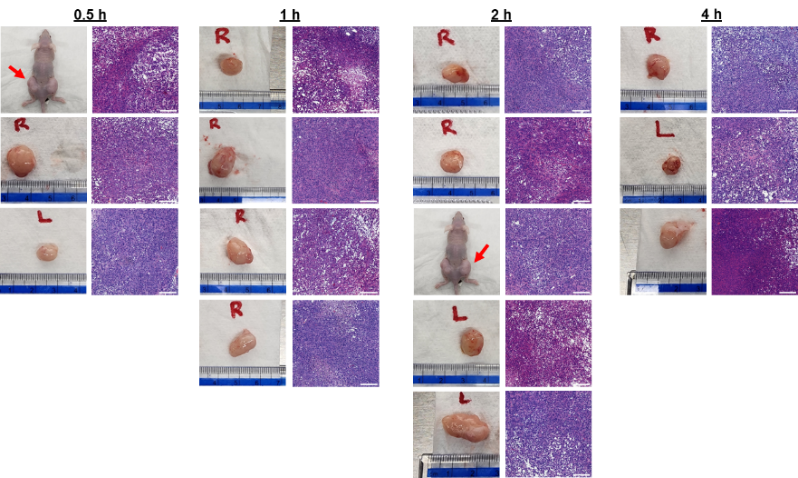
**

**Figure S13. Multi-dose in vivo tumors – treatment intervals.** Images of tumors upon resection on day 49 with representative micrographs of the tumors for the 0.5, 1, 2 and 4 h timepoints between the TRAIL injection and FUS exposure (scale bar = 200 µm). Tumor images were not acquired for one of the mice shown at 0.5 and 2 h.


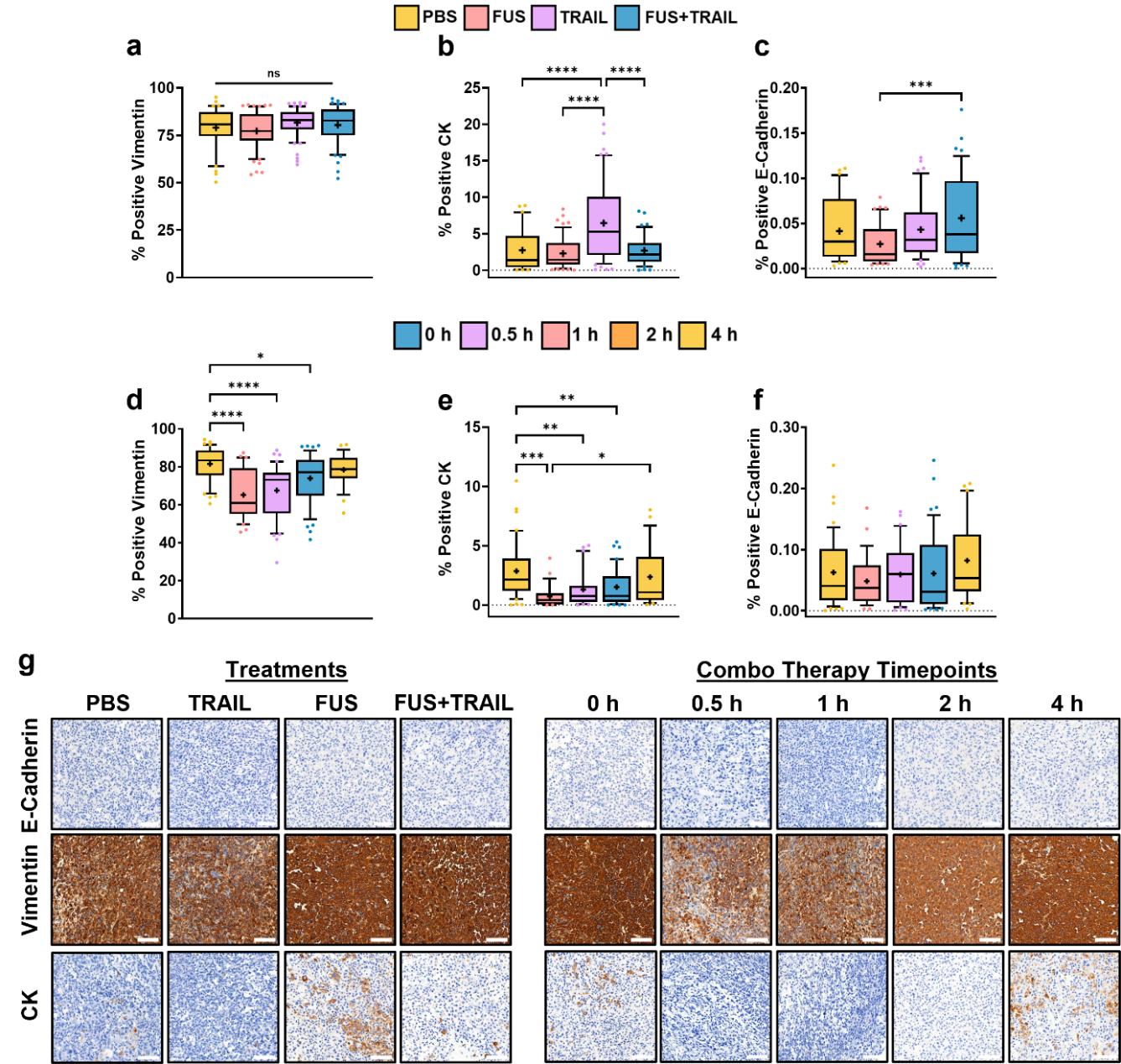


**Figure S14. IHC expression of EMT markers.** Summary data showing the mean percent positive tissue area of (a) vimentin, (b) CK and (c) E-cadherin expression for PBS, TRAIL, FUS or FUS+TRAIL therapy. Summary data showing the mean percent positive (a) vimentin, (b) CK and (c) E-cadherin expression for the treatment intervals between the TRAIL injection and FUS exposure. (g) Representative IHC micrographs (scale bar = 100 µm). Results correspond to the second in vivo study in Figures 6 and 7, one-way ANOVA (a-f). *p<0.05, **p<0.01, ***p<0.005, ****p<0.0001. Box and whisker plots showing individual data points outside of the 10-90th percentile with mean indicated as (+), median indicated as the solid horizontal line and outliers indicated as individual points (a-f).

Reference

[1] Á. Bartha, B. Győrffy, *Int J Mol Sci* **2021**, *22*, 2622.
